# Novel pyrene-calix[4]arene derivatives as highly sensitive sensors for nucleotides, DNA and RNA†

**DOI:** 10.1039/d3ra05696a

**Published:** 2023-09-13

**Authors:** Ivana Nikšić-Franjić, Benoit Colasson, Olivia Reinaud, Aleksandar Višnjevac, Ivo Piantanida, Dijana Pavlović Saftić

**Affiliations:** a Division of Physical Chemistry, Laboratory for Chemical and Biological Crystallography, Ruđer Bošković Institute Bijenička cesta 54 10000 Zagreb Croatia aleksandar.visnjevac@irb.hr; b Université de Paris - Laboratoire de Chimie et Biochimie Pharmacologiques et Toxicologiques, CNRS UMR 8601 45 rue des Saints Pères 75006 Paris France; c Division of Organic Chemistry and Biochemistry, Laboratory for Biomolecular Interactions and Spectroscopy, Ruđer Bošković Institute Bijenička cesta 54 10000 Zagreb Croatia dijana.pavlovic.saftic@irb.hr

## Abstract

Covalent functionalization of a calix[4]arene with one or two pyrene arms at one rim and two imidazoles at the opposite rim of the macrocyclic basket, yields fluorescent conjugates characterized by intramolecular pyrene-calixarene exciplex emission of a mono-pyrene conjugate, whereas the bis-pyrene derivative exhibits pyrene excimer fluorescence. The pyrene emission in these novel compounds is shown to be sensitive to non-covalent interactions with both mono- and polynucleotides. Pyrene-calixarene conjugates, acting as host molecules, strongly interact with nucleotides, as monitored by moderate emission quenching, reaching 0.1 μM affinities, comparable to some of the most effective supramolecular sensors for nucleotides. These compounds are efficiently inserted into ds-DNA/RNA grooves, with a high, 0.1–1 μM affinity, not influencing significantly any of the ds-polynucleotide native properties, whereby complete emission quenching allows the detection of DNA at nM concentration.

## Abstract 2

Pyrene-armed calix[4]arene conjugates non-covalently bind to DNA/RNA, as well as to mononucleotides, with an amazing affinity.

## Graphical abstract

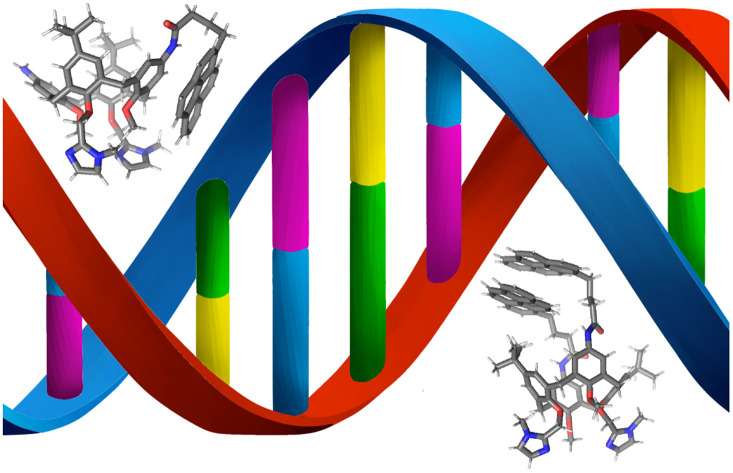

## Introduction

Calixarenes have been a subject of continuous scientific interest for several decades now, due to their extraordinary chemical versatility and plethora of application possibilities.^1–4^ We have developed three generations of specially designed calix[4]- and calix[6]arenes bearing multiple coordination arms or a capped unit (TMPA or TREN) grafted at either one of the rims.^5–8^ Some of these systems were recently studied by us as possible candidates for DNA/RNA binding in a frame of a wider project oriented towards the research of DNA/RNA recognition by specially designed calixarene derivatives.^9,10^ Development of new biomimetic systems capable of selective DNA/RNA binding is a subject of utmost importance for the development of new drugs, biosensors or tools for basic biochemical and biological studies.^11–13^

The covalently linked pyrene functional group, belonging to a group of polycyclic aromatic hydrocarbons (PAH), is especially interesting to us due to its unique properties like intense blue emission, high fluorescence quantum yield, long-lived singlet excited state, long emission lifetime (>100 ns), as well as its pronounced hydrophobicity. Many pyrene derivatives show intriguing biorelevant interactions, and, as strong chromophores and fluorophores, act as probes for biomacromolecules as, for instance, various DNA/RNA sequences.^14–18^ Pyrene can form variety of non-covalent interactions with DNA/RNA, like aromatic stacking intercalation into DNA/RNA, binding into the DNA minor groove *via* a combination of hydrophobic and edge-to-face aromatic interactions, or by forming pyrene excimer within the DNA minor or RNA major groove. Pyrene is also prone to form exciplex in combination with other chromophores.^19,20^ Due to these properties, pyrene is a sensitive fluorescent probe widely used for the characterization of different micro-heterogeneous systems.^21–27^ In a design of fluorescent sensors, an interesting approach is to attach two aromatic fluorophores close enough so that the electronic excitation of one ring can cause an enhanced interaction of its neighbour,^28^ leading to an excited-state dimer or excimer.^29^ In particular, calix[4]arene derivatives bearing two highly π-delocalized planar systems such as pyrenes, display an efficient excimer signal due to the intra- or intermolecular π–π interactions between the two pyrenes, and this excimer emission can be perturbed in presence of guest ions/molecules.^30^ For these reasons, pyrene-armed calix[4]arenes are widely used as fluorescent chemical sensors capable of selectively recognizing toxic cations,^31,32^ fluoride ions,^33^ and biologically and environmentally relevant anions such as cyanide, lactate, nitrite, nitrate and borate, and show potential analytical applications in environmental and biological areas.^34^

In order to enhance the capability of our calixarene based systems to bind to DNA or RNA, and to ensure efficient tracking of this binding by means of fluorometric methods, we have designed and synthesized a new series of calixarene derivatives, by grafting different fluorophores to the calixarene basket. We have previously demonstrated that particular cationic calixarene dimers bind into ds-DNA major groove, while monomeric cationic calixarenes revealed different DNA-binding profiles, likely to be positioned within the DNA minor groove.^10^ However, at that point, being focused on dimeric calixarenes, we did not study the interactions between the monomeric calixarenes and DNA/RNA systematically. Only recently we partially addressed that issue by studying interactions of neutral and cationic calixarenes with nucleotides and DNA, revealing the importance of positive charge for efficient binding to DNA.^9^

**Scheme 1:**
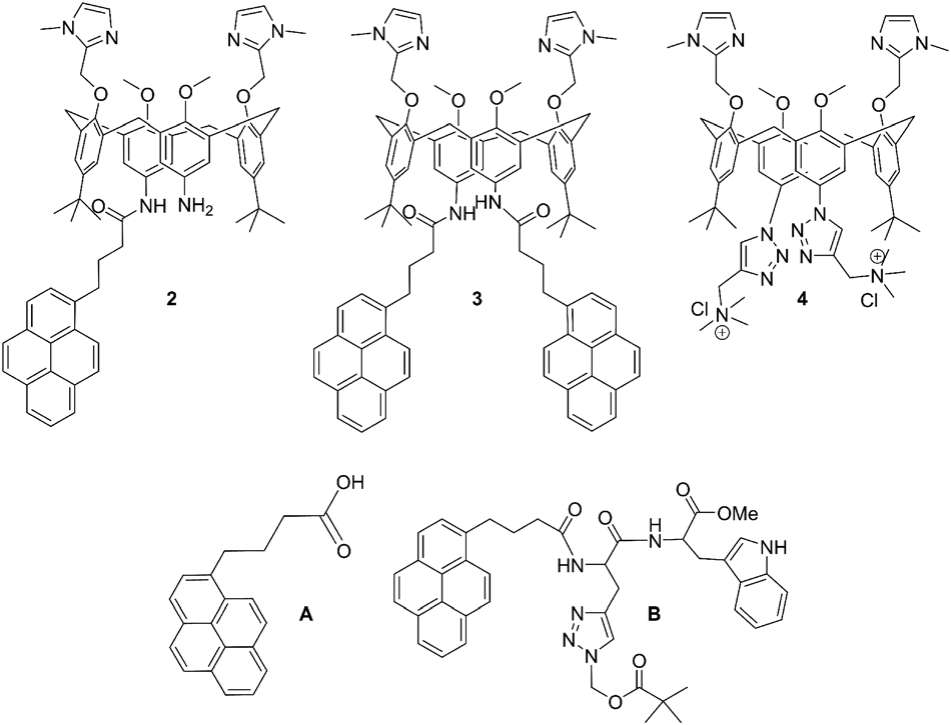
Molecular structures of novel calix[4]arene mono- (2) and bis-pyrene (3) derivatives studied in this work, and of referent compounds: previously studied analogue with two cationic triazole pendant arms (4),^35^ 1-pyrenebutyric acid (A) and pyrene-tryptophane peptide (B).^36^

In this paper, we report on the synthesis, photochemical and photophysical characterization of pyrene-calixarene conjugates 2 and 3 (Scheme 1), including quantum yield and fluorescence lifetime measurements, as well as the binding affinity of these compounds towards mononucleotides and DNA/RNA chains (ctDNA, p(dAdT)_2_, pApU, p(dGdC)_2_). The stability constants of the formed complexes were determined from UV-Vis and/or fluorescence titration data. The classical molecular dynamics simulations were carried out to gain a detailed insight into the structure of calixarene-pyrene conjugates. Circular dichroism (CD) spectropolarimetry was used to study conformational changes in the secondary structure of polynucleotides upon the addition of calixarenes, while the effect on the thermal stability of polynucleotides was investigated by calculating the melting temperature from thermal denaturation curves. To the best of our knowledge, this is the first study on the recognition of mono- and polynucleotides by calixarene-pyrene conjugates.

## Results and discussion

### Synthesis

**Scheme 2:**
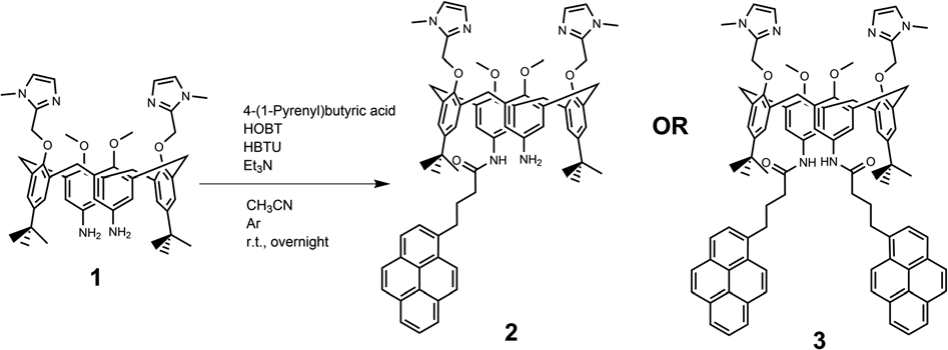
Synthesis of calix[4]arene-pyrene conjugates 2 and 3 from 1.

Novel compounds were synthesized as described in Scheme 2, by a one-step coupling of pyrene–butyric acid activated by HOBT/HBTU with calix(4)arene diamine 1,^35^ whereby variation of added pyrene-reagent resulted in mono- (2) or bis-pyrene (3) products, both with *ca.* 40% yield. Structures of obtained final compounds were fully characterized by NMR and HRMS analysis (ESI Fig. S1–S6†).

### Spectrophotometric characterisation of 2 and 3

Compounds 2 and 3 are poorly soluble in water, hence for the purpose of a biorelevant study, we have prepared their stock solutions in DMSO (5 mM), which were stable at 4 °C. For all further experiments, aliquots of stock solutions were diluted in buffer (sodium cacodylate buffer, *I* = 50 mM, pH 7.0) prior to measurement. It should be noted that at pH 7, two imidazole units are partially protonated (p*K*_a_ = 7), thus compounds have a net +1 positive charge. The absorbances of aqueous solutions of 2 and 3 were proportional to their concentrations up to *c* = 2 × 10^−5^ M (ESI, Fig. S7†). The absorption maxima and their corresponding molar extinction coefficients are given in Table 1.

**Table 1:** Electronic absorption and emission data of: 2, 3, A a (1-pyrenebutyric acid) and B (pyrene-tryptophane peptide)^a^

Comp.	*λ*_max_/nm	*ε*/dm^3^ mol^−1^ cm^−1^	*Φ*_f_ g	*λ*_exc_ b/nm	*λ*_em_/nm	τ^c^/ns (degassed)	*χ*2
2	350^d^	31 440^d^	26.49 ± 0.02^d^	340	Water, 475	2.13 (15.62%)^d^	1.178^d^
						12.66 (45.42%)^d^	
						47.23 (38.97%)^d^	
					DMSO	0.67 (5.61%)^e^	1.117^e^
						4.51 (9.87%)^e^	
						47.60 (84.52%)^e^	
	346^e^	32 363^e^	28.76 ± 0.02^e^		Water, 400	0.14 (6.64%)^d^	1.118^d^
						4.40 (7.85%)^d^	
						120.79 (85.51%)^d^	
					DMSO	4.98 (11.69%)^e^	1.137^e^
						20.92 (58.55%)^e^	
						68.89 (29.75)^e^	
3	350^d^	30 299^d^	50.05 ± 0.02^d^	340	Water, 475	6.84 (8.21%)^d^	1.101^d^
						26.69 (43.90%)^d^	
						64.65 (47.89%)^d^	
					DMSO	12.89^e^^,^^f^	1.228^e^
						49.57^e^^,^^f^	
	346^e^	43 173^e^	31.56 ± 0.02^e^		Water, 400	0.57 (8.56%)^d^	1.180^d^
						3.60 (24.84%)^d^	
						14.95 (28.39%)^d^	
						84.98 (38.21%)^d^	
					DMSO	10.90 (11.91%)^e^	1.161^e^
						39.58 (77.57%)^e^	
						96.21 (10.52%)^e^	
A a	342	62 596 ± 1248^d^	0.15 ± 0.02^d^	340	398	2.5 (1%)^d^	1.060
						100.3 (99.2%)^d^	
B a	350	25 012 ± 638^d^	0.02 ± 0.005^d^	340	472	11.6 (62%)^d^	1.223
						36.4 (37%)^d^	

a See ref. 36.

b Samples were excited by pulsing diode at 340 nm. The measurements were performed three times and the average values are reported. The associated errors correspond to the maximum absolute deviation.

c Solutions were purged by argon.

d Done in water.

e Done in DMSO.

f Negative pre-exponential factor, without relative representation characteristic for excimer formation.

g Absolute fluorescence quantum yield was determined by integrating sphere SC-30, Edinburgh Inst., for argon purged solutions, by *λ*_exc_ = 353 nm.

**Fig. 1:**
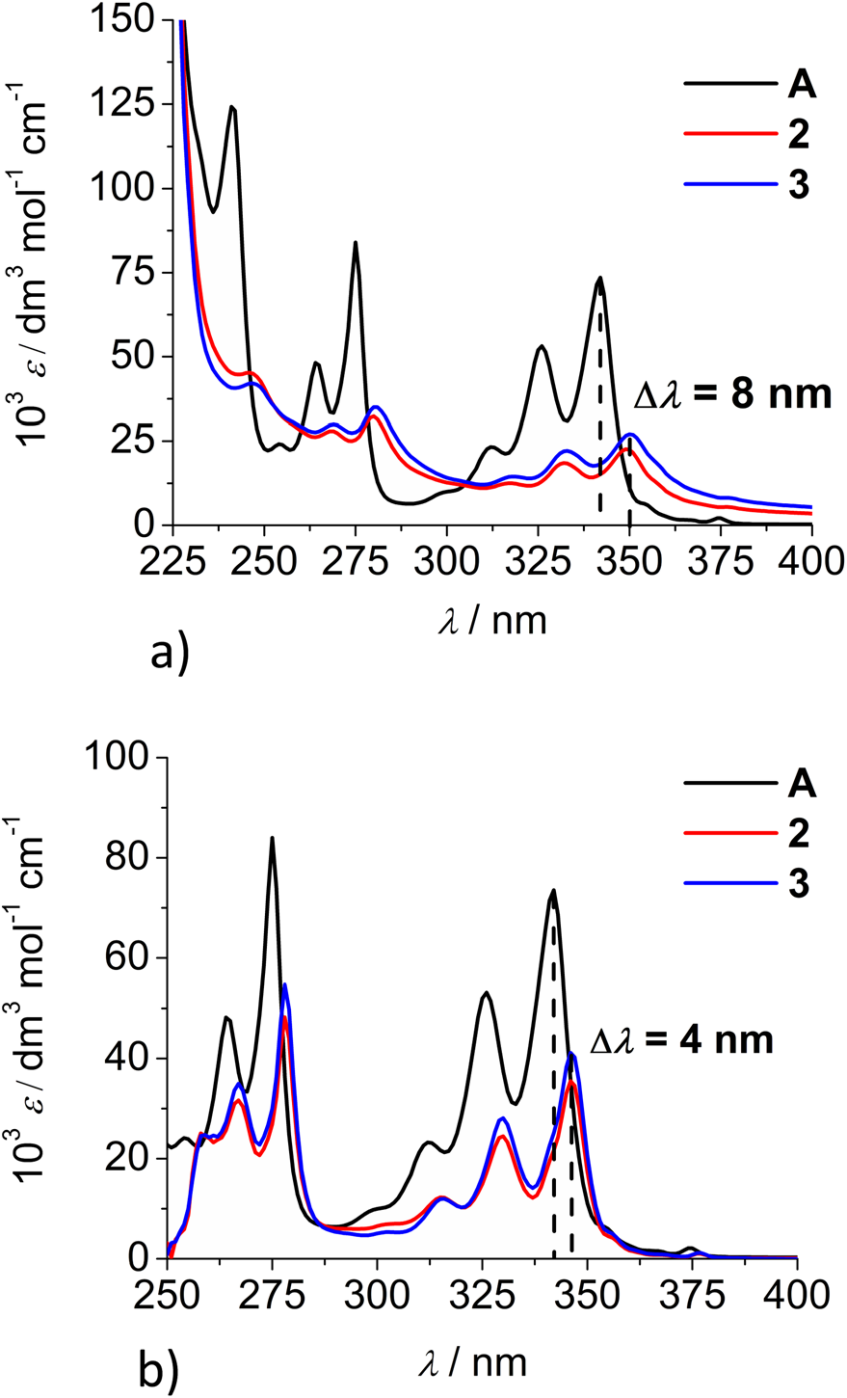
Comparison of UV/Vis spectra: A (1-pyrenebutyric acid),^36^2 and 3 at concentration *c* = 1 × 10^−5^ M in: (a) water, (b) DMSO. The Δ*λ* values are shown only for 342 nm maximum of A but bathochromic shift is present for complete spectra of 2 or 3.

Comparison of the UV/Vis spectra (Fig. 1a) reveals a strong bathochromic and hypochromic effect of conjugates 2 and 3 in respect to the referent chromophore (1-pyrenebutyric acid, A). As A is free of any hydrophobic or aromatic interaction, obtained spectra strongly suggest intramolecular aromatic stacking interactions in 2 and 3. The analogous UV/Vis spectra collected in DMSO (Fig. 1b) reveal less pronounced but still present bathochromic and hypochromic effects in 2 and 3, thus demonstrating that DMSO did not completely cancel intra- or inter-molecular interactions of pyrene in 2 and 3.

**Fig. 2:**
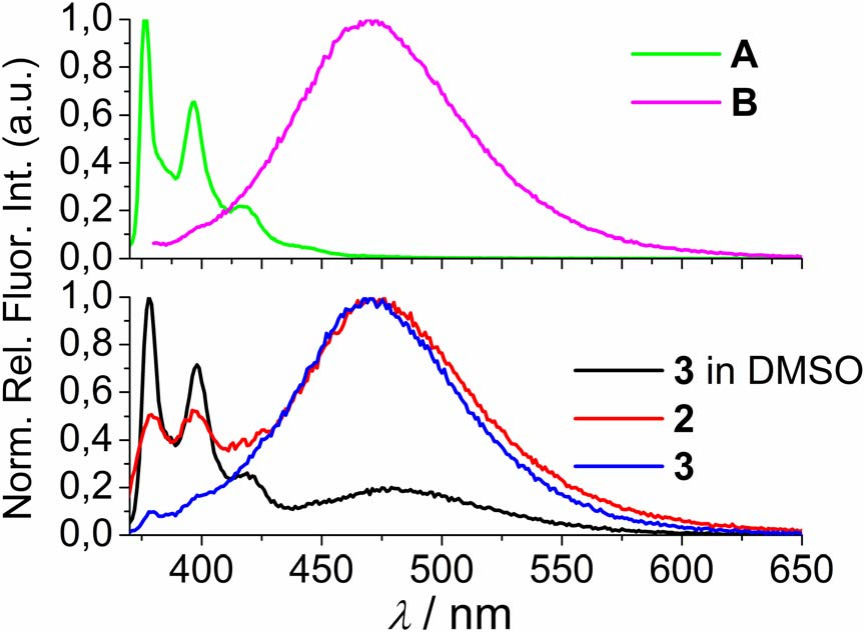
(Top) 1-pyrenebutyric acid A (*λ*_exc_ = 342 nm) and peptide B (*λ*_exc_ = 352 nm) in water (0.01% DMSO). (Bottom) Normalised fluorescence spectra of 2 (*λ*_exc_ = 352 nm, water), 3 (*λ*_exc_ = 350 nm, water) and 3 (*λ*_exc_ = 350 nm, DMSO). Normalisation was performed by dividing a complete spectrum by the maximal intensity of emission.

Both studied derivatives show fluorescence emission in aqueous and DMSO solutions (Fig. 2), with emission intensities proportional to their concentration up to *c* = 2 × 10^−6^ M (ESI, Fig. S8†), thus excluding intermolecular interactions. Intriguingly, 2 and 3 show the strongest bathochromic shift of emission maximum (+70 nm) with respect to the referent 1-pyrenebutyric acid (A), and similar to the previously noted effect of referent peptide B (Fig. 2, top), suggesting the formation of either pyrene excimer or exciplex between pyrene and calixarene aromatic moieties.^36^ The temperature variation induced pronounced changes in the fluorescence spectra of the conjugates 2 and 3 (ESI, Fig. S9 and S10†), confirming intramolecular interactions of the chromophores. However, mono-pyrene analogue 2 showed minor peaks in the 370–430 nm range (in a good agreement with spectrum of A), suggesting that part of the pyrene in 2 is freely exposed to water, thus not in the excimer/exciplex form. Adversely to 2, bis-pyrene analogue 3 reveals a spectrum in water with only a single maximum at 470 nm, whereas its fluorescence spectrum in DMSO coincides to a great extent with the spectrum of reference A, with only a minor emission at 480 nm, pointing out that even in DMSO not all pyrene chromophores in 3 are void of excimer/exciplex interactions.

At this point, question arose whether the observed strongly shifted emissions of 2 and 3 are due to the pyrene excimer formed between two pyrene units or due to the pyrene-calixarene exciplex formation. For mono-pyrene derivative 2, linear dependence of emission intensity on *c*(2) excluded intermolecular stacking of two molecules of 2 for pyrene excimer, thus observed effect is likely the same as noted for referent peptide B:^36^ formation of intramolecular pyrene exciplex with another aromatic unit of the same molecule.

Nevertheless, the bis-pyrene-calix[4]arene 3 strong emission can be attributed to either: (a) intramolecular pyrene excimer emission,^14*a*,37−40^ or (b) pyrene exciplex emission,^40,41^ in which a heterodimeric exciplex of pyrene and aromatic rings of calixarene core or imidazole(s) is intramolecularly formed.

If pyrene forms intramolecular aromatic stacking interactions with imidazole, the stability and emission response of such exciplex should be strongly affected by protonation/deprotonation of imidazole (p*K*_a_ 7). Emission spectra of 2 and 3 collected at pH = 6–8.5 (ESI, Fig. S11†) reveal only a minor difference in intensity but the shape of the emission spectrum remains unchanged, suggesting that imidazole is not in interaction with pyrene(s).

In order to obtain a deeper insight into the photophysical processes in solutions of 2 and 3, we have collected time-resolved fluorescence decay data (*τ* in Table 1, ESI, Fig. S12 and S13†) and compared them with two referent compounds, A (free, non-stacked pyrene) and B (pyrene exciplex with another aromatic unit).^36^ The data were collected at two emission wavelengths: 475 nm (characteristic for excimer/exciplex) and 400 nm (characteristic for non-stacked pyrene), and in two solvents, DMSO and water (only the latter stimulates aromatic stacking and hydrophobic interactions).

Analysis of time-resolved fluorescence decay data (*τ* in Table 1) shows (at 400 nm) the presence of long-lived emitting species in all cases, attributed to the free pyrene fluorophore (in comparison to reference A), thus not in the interaction with another aromatic moiety (*e.g.* calixarene or another pyrene). Data collected at 475 nm reveal the presence of several shorter-living emissive species, which are by comparison to reference B and literature data^37^ attributed to the pyrene excimer or exciplex. In an aqueous medium, analogues 2 and 3 show a similar distribution of three species, whereby two values of similar magnitude dominate (12.66 and 47.23 ns for 2; 26.69 and 64.65 ns for 3). In DMSO, at 475 nm both 2 and 3 show very similar dominant values, 47.60 or 49.57 ns, respectively, pointing out that upon exclusion of water, one dominant type of pyrene stacked with another aromatic moiety remains. For mono-pyrene analogue 2, this can only be a pyrene stacked with a calixarene aromatic system (exciplex similar to reference B), and an almost identical *τ* value observed for bis-pyrene 3 suggests similar intramolecular interaction. However, in water, bis-pyrene 3 might form, besides an exciplex, an intramolecular pyrene excimer with calixarene aromatics. Thus in both, 2 and 3, the pyrene fluorophore(s) is/are in contact with the calix[4]arene core and, consequently, the fluorescence of 2 and 3 should be very sensitive to any binding event with DNA/RNA targets.

### Molecular modelling

In order to clarify whether the dominant interaction is formation of excimer (stacking between two pyrene units) or exciplex (pyrene–benzene π–π interaction), we have performed molecular dynamics simulations of 2 and 3. Attaching one or two relatively large pyrene groups to calixarene scaffold can induce conformational changes in solution due to a free rotation of these “legs” around the linkers (in 2 and 3 these linkers contain three methylene groups). Some previous studies have revealed the possibility of an inclusion of single pyrene unit into the calixarene^25,28^ or cyclodextrine^34^ macrocyclic cavity which significantly influences the fluorescence sensing.

**Fig. 3:**
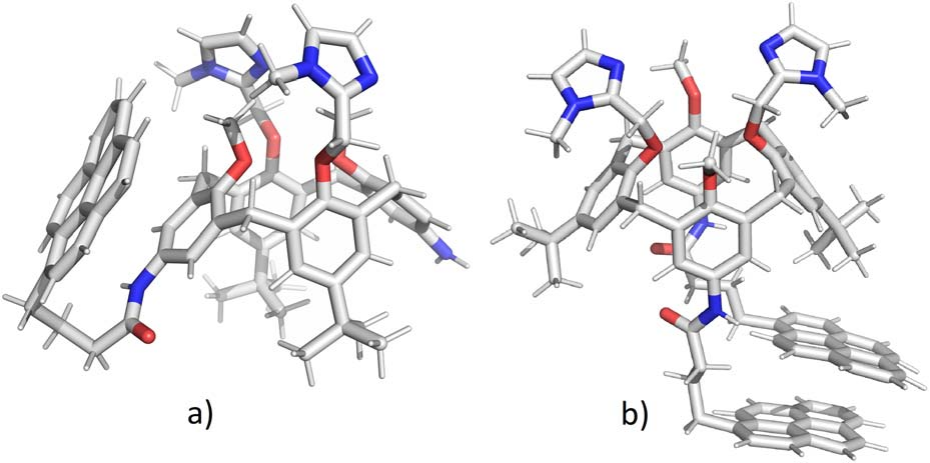
Structures of calixarenes 2 (a) and 3 (b) after 10 ns at *T* = 300 K. Water molecules are omitted for clarity.

The initial structures of calixarenes 2 and 3 for molecular dynamics simulations were prepared in cone conformation with pyrene rings stretched below the calixarene lower rim. After 10 ns MD simulation (Fig. 3a) in water, in conjugate 2 pyrene forms aromatic stacking interactions with the benzene ring from the calixarene basket, which supports the hypothesis of exciplex formation (*vide supra*). The structure of its bis-pyrene analogue 3 after 10 ns MD simulation (Fig. 3b) was characterized by two mutually parallel pyrene arms with average distances between corresponding carbon atoms in the range of 3–4 Å, supporting pyrene excimer formation. Hence, although both conjugates have the emission maxima at 475 nm, the origin of this emission can be attributed to non-covalent interactions between different moieties.

The encapsulation of pyrene fluorophore(s) between pendant “legs” or into the cavity was not observed because of steric hindrances. Peptide bond is planar without possibility of rotation limiting the possibility of bending a calix-pyrene linker. Furthermore, the energy penalty for reorientation of functional groups and extending the calix[4]arene cavity for inclusion of pyrene would be too large. Hence, the formation of exciplex for 2 is achieved on the outside of the cavity meaning that pyrene is partially exposed to water molecules which correlates with the experimental minor fluorescence peaks at 370–430 nm for free pyrene.

Both 2 and 3 are versatile macrocycles with plethora of possibilities to accommodate guests like ions, small molecules, solvents, *etc.* Conformations of the ligands are expected to be altered upon the complexation since the upper and lower rim groups orientate inwards to encapsulate the guest in the binding site, while in the host–guest complex, due to variety of non-covalent interactions induced by inclusion, the structure of calixarene becomes rigidified upon inclusion. In other words, aromatic stacking interactions as well as hydrogen bonds of electron-donating atoms (*e.g.* oxygen and nitrogen) with guest molecules may determine the structure and size of the binding site.

Due to the steric hindrance of methylimidazoles on the upper and pyrene and *tert*-butyl groups on the lower rim, the binding sites at the two rims are asymmetric and the relative orientation of the functional subunits is different in conjugates 2 and 3. 2 forms stacking interactions with benzene ring causing significantly larger distance between methylimidazoles as compared to 3. For instance, the distances between oxygen –OCH_2_Im atoms for calixarene 2 are 5.0 Å and 3.5 Å for 3. On the other hand, the methoxy oxygen atoms are significantly closer in 2 as compared to 3: *d*(2) = 3.6 Å, *d*(3) = 5.7 Å. The distances between free N-atoms of methylimidazole are approximately the same for 2 and 3, around 5.2 Å. On the lower rim, in the binding site of 2 the N–N amino groups are separated for approximately 11.5 Å, while for 3 peptide bond oxygen atoms are oriented towards each other due to π–π interaction between pyrenes: *d*(O–O) = 3 Å. *Tert*-butyl groups are practically parallel in mono-pyrene derivative while for bis-pyrene derivative they are oriented outside of the calix-rim to reduce steric hindrance. Considering all these structural effects, the upper rim of calixarene 2 is narrow and the lower rim is extended, while for 3 stacking of pyrenes reduces the size of binding site and induces the rotation of benzene and imidazole moieties on upper rim giving a wider binding site.

Calculations were also performed for cationic +1 forms of 2 and 3 since at pH = 7 two imidazole moieties are partially protonated forming an imidazolium ion. The MD simulations started from the same initial structures as for the neutral conjugates, but with a proton added to one of the free N-atoms of imidazoles. The results revealed that protonation of imidazoles does not induce significant conformational changes when considering the orientation of pyrene subunit relative to calixarene scaffold. This is especially true for bis-pyrene derivative where the protonation of imidazole cannot disrupt very strong hydrophobic interactions between pyrenes on the opposite rim. However, the positively charged imidazolium arm of mono-pyrene tends to move towards π-electron rich pyrene subunit during the simulation but the relative orientation of rings is not so favourable for aromatic stacking interactions, as for pyrene-benzene ring. This supports the emission spectra of 2 and 3 which change only slightly when changing pH from 6 to 8.5 (ESI, Fig. S11†) suggesting that imidazolium is not in direct interaction with pyrene.

### Non-covalent interactions of 2 and 3 with various mononucleotides (AMP, CMP, GMP, and UMP) and ds-DNA/ds-RNA

#### Spectrophotometric titrations

As many pyrene derivatives exhibit captivating biorelevant interactions, and, as strong chromophores and fluorophores, act as probes for various DNA/RNA sequences, we performed preliminary studies on interactions of derivatives 2 and 3 with several mononucleotides and with the most commonly used ds-DNA representatives – naturally isolated calf thymus (ct)-DNA characterized by typical B-helix and approximately equimolar number of G–C and A–T base pairs, synthetic polynucleotides poly(dAdT)_2_, and poly(dGdC)_2_ also having B-helical structure, as well as with the analogous synthetic ds-RNA representative: poly A–poly U, characterized by A-helical structure.

In general, the addition of any mononucleotide or ds-DNA/ds-RNA to 2 or 3 resulted only in moderate hypochromic effect in 2 or 3 UV/Vis spectrum (ESI, Fig. S15–S22 and S26–S33†), typical for the engagement of the pyrene chromophore in aromatic stacking interactions.^37^ UV/Vis spectra changes of both studied conjugates show no selectivity towards any of the mono-/polynucleotides employed (ESI, Fig. S15–S22 and S26–S33†).

The titrations result with dominant changes of 2 and 3 UV/Vis spectra at excess of each studied dye to mono-/polynucleotide, which is unfavourable for the determination of single molecule interaction with DNA/RNA. Namely, at such conditions, dyes tend to aggregate along the polynucleotide,^42^ which can result in the apparent increase of binding affinity caused by monitoring the spectroscopic changes of two events (de-aggregation and nucleotide binding) simultaneously.

**Fig. 4:**
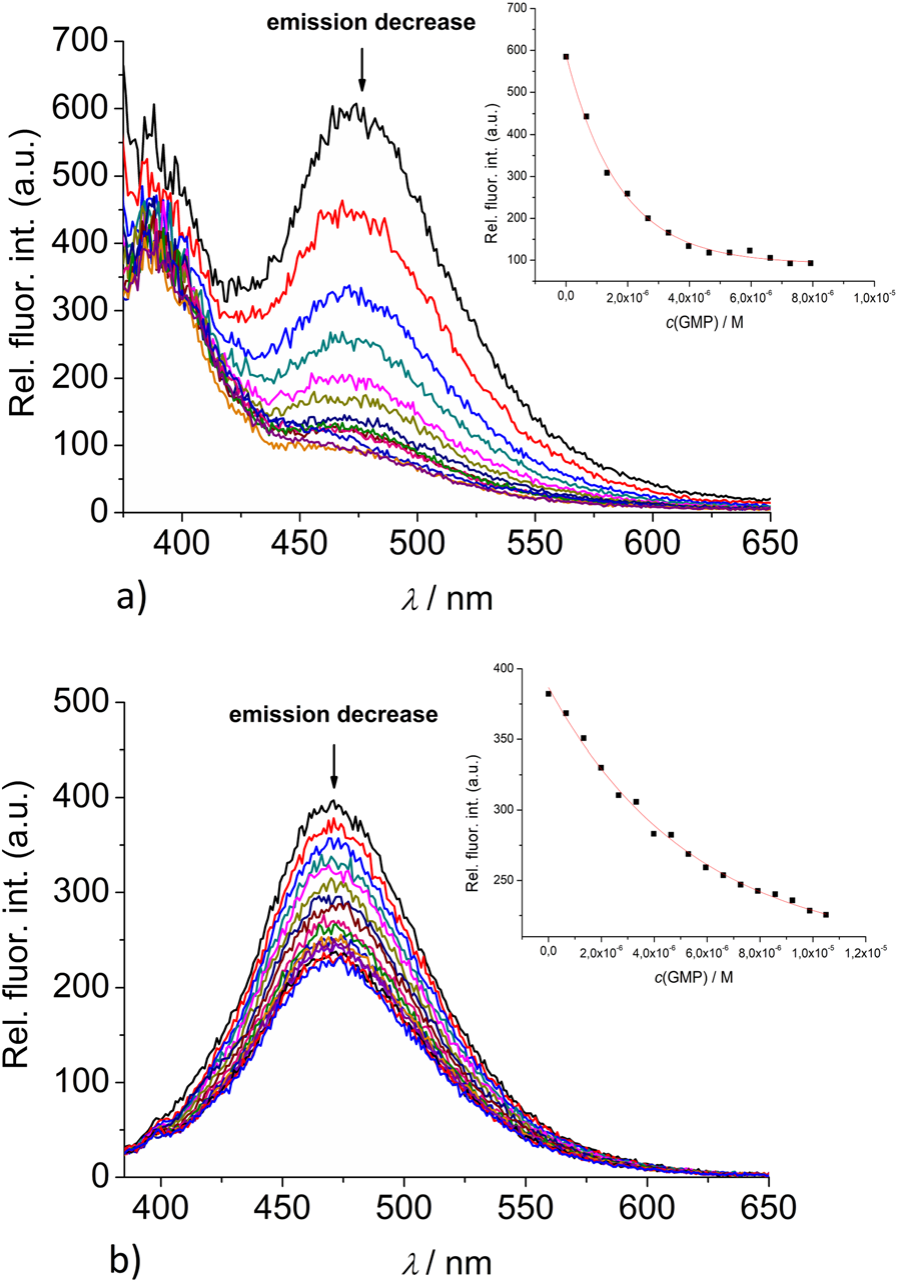
Changes in the fluorescence spectra of: (a) 2 (*c* = 5 × 10^−7^ M) and (b) 3 (*c* = 1 × 10^−6^ M), upon titration with GMP (*c* = 1 × 10^−3^ M) at *λ*_exc_ = 350 nm. (Insets) Dependence of 2 and 3 emission intensity at 475 nm on *c*(GMP). Done at pH 7.0, sodium cacodylate buffer, *I* = 0.05 M.

**Fig. 5:**
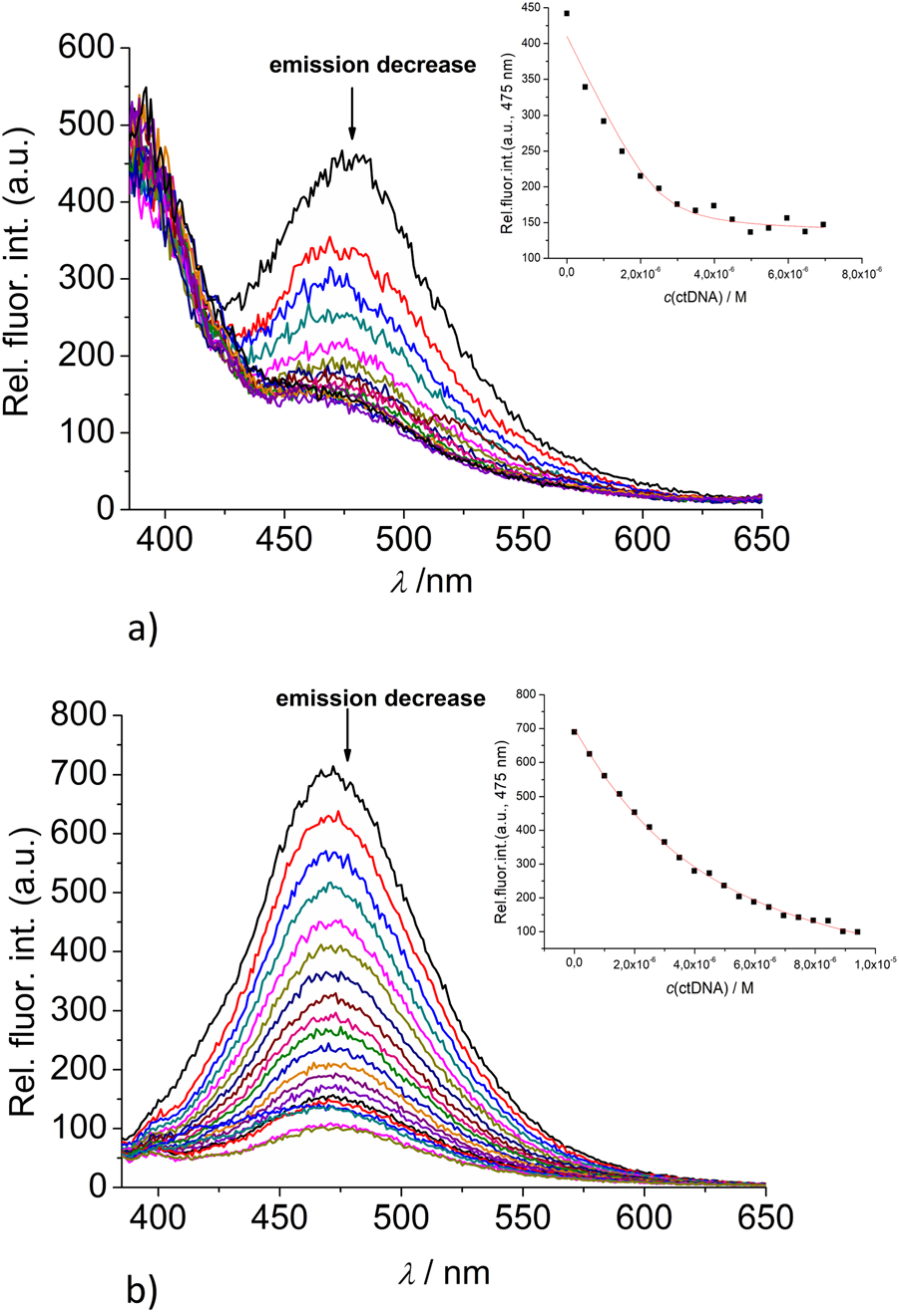
Changes in the fluorescence spectra of: (a) 2 (*c* = 5 × 10^−7^ M) and (b) 3 (*c* = 5 × 10^−7^ M) upon titration with ctDNA (*c* = 1 × 10^−3^ M) at *λ*_exc_ = 350 nm. (Insets) Dependence of 2 and 3 emission intensity at 475 nm on *c*(ctDNA). Done at pH 7.0, sodium cacodylate buffer, *I* = 0.05 M.

Fortunately, strong fluorescence of 2 and 3 allowed us to perform fluorometric titrations at much lower molar ratios *r* = [dye]/[polynucleotide] (*r* ≤ 1), and at a concentration of dye at which no intermolecular aggregation is present (5.0 × 10^−7^ M). The incubation after every addition was two minutes. It was determined experimentally that after one minute upon each aliquot addition no further changes in spectrum occurred. Thus, double time (two minutes) was considered adequate for the incubation to ensure thermodynamic equilibrium. These conditions allowed accurate processing of titration data by non-linear fitting procedure to Scatchard equation,^43–45^ or for the mononucleotides the best fit was obtained for 1 : 1 stoichiometry of dye/NMP complex formed (Table 2). The addition of any mononucleotide or ds-DNA/ds-RNA to 2 and 3 resulted in quenching their emission (Fig. 4 and 5; ESI; Fig. S23–S25 and S34–S36†).

**Table 2:** Binding constants (log *K*^a^) and emission quenching efficiency (ΔInt^b^) for 2 and 3 with mononucleotides and ds-DNA/ds-RNA determined fluorometrically. Done in sodium cacodylate buffer (pH = 7.0, *I* = 0.05 M). Data given for referent 1 and 4 are published previously^9^

	1 c	2	3	4 c
CMP	—	5.8 (0.7^b^)	5.6 (0.3^b^)	3.1 (0.2^b^)
AMP	5.0 (0.8^b^)	5.9 (0.7^b^)	5.6 (0.3^b^)	4.3 (0.9^b^)
UMP	4.1 (0.6^b^)	5.7 (0.2^b^)	5.0 (0.7^b^)	2.5 (0.4^b^)
GMP	4.7 (0.8^b^)	5.8 (0.2^b^)	5.3 (0.6^b^)	4.8 (0.6^b^)

ctDNA	—	7.6 (0.3^b^)	6.2 (0.1^b^)	5.2^c^
p(dAdT)_2_	—	7.4 (0.2^b^)	6.6 (0.3^b^)	5.0^c^
pApU	—	6.4 (0.2^b^)	7.2 (0.2^b^)	5.1^c^
p(dGdC)_2_	—	5.7 (0.3^b^)	6.9 (0.2^b^)	5.0^c^

a The best fit of experimental data was obtained for 1 : 1 stoichiometry of dye/NMP complex or for DNA/RNA by processing of titration data by means of Scatchard equation gave values of ratio *n*[bound dye]/[DNA/RNA] = 0.1 and 0.2, for easier comparison all log *K* values were re-calculated for fixed *n* = 0.2.

b ΔInt = Int(100% complex)/Int_0_.

c Published data.^9^ For DNA/RNA log *K* values estimated from competition experiments.

Upon addition of nucleotide or DNA/RNA, emission of mono-pyrene 2 was strongly quenched only at exciplex maximum at 475 nm, while emission at 390 nm (attributed to the free pyrene) only negligibly changed. Such changes would imply that a complexation event causes major disruption of pyrene-calixarene exciplex. The pyrene-excimer emission of bis-pyrene analogue 3 was partially quenched by nucleotides (Fig. 4) and completely abolished by ds-DNA/RNA (Fig. 5).

Detailed analysis of titration experiments reveal that the binding constants obtained by UV/Vis titrations (ESI; Table S2,† log *K*) differ somewhat in comparison to those resulting from fluorometric titrations (Table 2), likely due to the unfavourably high concentrations of 2 or 3, causing partial aggregation of dye at high rations *r*[dye]/[nucleotide or DNA]. Thus, the log *K* values obtained by fluorometric titrations are more reliable and could be compared with data previously obtained for 1 and 4.

The comparison of obtained data for 2 or 3 with those previously published^9^ for referent calixarene 1 and its permanently 2+ charged analogue 4, reveals several intriguing points. The pyrene-containing calixarenes 2 and 3 bind nucleotides with similar affinity (Table 2), but at least an order of magnitude stronger in comparison to referent 1 or permanently 2+ charged analogue 4. Particularly significant differences are observed for pyrimidine nucleotides (UMP, CMP), which 2 and 3 bind two or even three orders of magnitude stronger than 1 or 4. Taking into account that only 2 and 3 contain pyrene moieties, such differences strongly support aromatic stacking interactions between pyrene and nucleobase as the main reason for increased affinity as compared to 1 or 4; agreeing well with previous binding studies of pyrene-containing derivatives with nucleotides under comparable conditions, giving values of log *K* ∼ 2.^46^ Summarised results are implying a general rule for hereby studied series of calixarenes: grafting the pyrene subunits to calixarene analogue 1 increases the stability constant of the calixarene – mononucleotide non-covalent complex by a factor of 10–100.

The interactions of studied calixarenes with ds-DNA or ds-RNA are significantly different in comparison to those with nucleotides, by means that nucleotides “insert” into the structure of calixarenes, whereas calixarenes “insert” into grooves of polynucleotides.^10^ We showed previously^9^ that referent calixarene 1 does not interact with DNA/RNA unless a positive charge is introduced into the structure, as is the case in the analogue 4. However, hereby obtained results (Table 2) reveal that pyrene-containing conjugates 2 and 3 strongly interact with DNA/RNA even if they have no permanent positive charge whatsoever.

The question remains which type of interaction with DNA/RNA pyrenes attached to 2 and 3 provide: (i) intercalation between DNA-base pairs; or (ii) hydrophobic-driven insertion into DNA/RNA grooves with possible edge-to-face aromatic interactions between pyrene and nucleobases. Pyrene UV/vis and fluorescence spectra cannot give accurate cues on this aspect, thus more structurally informative methods are needed. Insufficient solubility of 2 and 3 in water, however, hampered NMR experiments with oligomeric DNA.

### Circular dichroism (CD) experiments

To gain an additional insight into the structural mode of binding of our conjugates 2 and 3 to ds-DNA and ds-RNA we have employed circular dichroism (CD) spectroscopy. CD spectroscopy is a useful analytical tool to study the interactions of small molecules with chiral macromolecules such as DNA,^47^ since it can provide information on the mode of binding to a polynucleotide, with distinctive spectral differences for intercalators and groove-binding derivatives.^48,49^

Both 2 and 3 are achiral with no measurable CD bands within the 240–400 nm range (ESI, Fig. S37†), thus not interfering with the CD bands of DNA/RNA in the 240–290 nm range, nor with the eventually induced CD bands of the pyrenes, which could appear at >300 nm upon binding to ds-DNA/RNA.

The results of the titration data (ESI, Fig. S38–S43,† the molar ratios *r*(dye/DNA) = 0.1, 0.3 and 0.5) reveal the negligible influence of the studied calixarene derivatives 2 and 3 on the helicity of studied polynucleotides, thus excluding classical intercalation of pyrene between base pairs.^48^ Moreover, in the range >300 nm no induced CD bands were observed, suggesting that upon binding to DNA/RNA, pyrene moieties of 2 and 3 are not uniformly oriented in respect to the polynucleotide chiral axis. Thus, obtained results support the non-specific binding of 2 and 3 within DNA/RNA grooves.

### Thermal denaturation of ds-DNA/ds-RNA

Thermally-induced dissociation of the ds-polynucleotides into two single-stranded polynucleotides occurs at a well-defined temperature (*T*_m_ value), thus being used for the characterization of various ds-DNA or ds-RNA-related processes. Non-covalent binding of small molecules to ds-polynucleotides usually increases the thermal stability of the ds-helices, thus resulting in an increased *T*_m_ value, and this increase (Δ*T*_m_) can (in corroboration with other methods) be related to the various binding modes.^50^ For example, most pyrene analogues by intercalating into ds-DNA/ds-RNA, cause stabilization for Δ*T*_m_ > +5 °C due to aromatic stacking interactions with polynucleotide base pairs, whereas the binding of pyrenes within the polynucleotide groove, usually driven mostly by a hydrophobic effect accompanied by weak H-bonding, should have a negligible stabilizing outcome (Δ*T*_m_ < 2).^51^ In our previous study of referent calixarenes 1 and 4,^9^ we demonstrated that electrostatic interactions are controlling the thermal stabilization effect, neutral calixarene 1 having no impact on ds-DNA/RNA thermal denaturation, unless it is in cationic form (achieved by adding positively charged substituents – analogue 4).

The thermal denaturation results revealed that adding 2 or 3 did not stabilize ct-DNA or ds-RNA against thermal denaturation (ESI; Table S3, Fig. S44 and S45,† the molar ratios, *r*(dye/DNA) = 0.2 and 0.3) which supports the hypothesis that these conjugates bind to the grooves, rather than by intercalation.^17*a*,50^

## Conclusions

Newly designed and prepared pyrene-containing calix[4]arene conjugates 2 and 3 are characterized by studying their distinct intramolecular aromatic stacking interactions in an aqueous medium. Strong bathochromic shift of mono-pyrene derivative 2 fluorescence emission relative to the free pyrene maximum is attributed to the formation of pyrene-calixarene exciplex, whereas a similar effect observed for bis-pyrene derivative 3 is a consequence of the intramolecular pyrene-excimer formation.

Since the emissions of 2 and 3 are controlled by intramolecular aromatic stacking interactions, it is necessarily exceedingly sensitive to any kind of interaction with other molecules, which would cause a conformational change of calixarene and/or modify the microenvironment around pyrene fluorophores.

Pyrene-calix[4]arene conjugates 2 and 3 strongly interact with mononucleotides, combining hydrophobic interactions of parent calix[4]arene 1 (ref. 9) with additional aromatic stacking interactions of pyrene, thus reaching the 0.1 μM affinities in water, which is comparable to some of the most effective supramolecular sensors for nucleotides.^37–39^ Although no selectivity between recognition of various nucleotides by 2 and 3 was observed, these systems, with appropriate additional structural modifications to enable selective recognition of different mononucleotides, thanks to their extraordinary efficient nucleotide binding capacity, can easily evolve to new lead compounds.

Novel pyrene-calix[4]arene conjugates 2 and 3 bind into ds-DNA or ds-RNA grooves non-specifically, with high (0.1–1 μM) affinity, not influencing significantly any of the ds-polynucleotide native properties (secondary structure and thermal denaturation point). The intrinsic emission of 2 and 3 is sturdily quenched upon binding, thus allowing the detection of interaction with DNA at as low as nM concentration. These findings invite for further biological studies, focusing on the intrinsic cytotoxicity of 2 and 3, as well as the photo-induced cytotoxicity of pyrene tether, well-known for ability of singlet oxygen sensitization.^37^

## Experimental

### Chemicals for synthesis and physicochemical investigations

Chemicals for synthesis: 4-(1-pyrenyl)butyric acid (Alfa Aesar, 97%), triethylamine (Et_3_N) (Sigma Aldrich, 99%), hydroxybenzotriazole (HOBt) (Sigma Aldrich, 97%), 2-(1*H*-benzotriazole-1-yl)-1,1,3,3-tetramethyluronium hexafluorophosphate (HBTU) (Alfa Aesar, 98%), Silica gel 60, PF_254_ for preparative thin layer chromatography (Merck).

Nucleotides (AMP, GMP, UMP and CMP) were purchased from Sigma, dissolved in stock solutions of *c* = 0.01 M and used. Polynucleotides were purchased as noted: poly dAdT–poly dAdT, poly A–poly U, poly dGdC–poly dGdC, (Sigma), calf thymus (ct)-DNA (Aldrich) and dissolved in sodium cacodylate buffer (*I* = 0.05 M, pH = 7.0), as described by producer. The presence of double stranded helix was confirmed by single, well-defined transition in the thermal denaturation experiment, giving *T*_m_ value agreeing well with the literature; as well as by collecting CD spectra of free ds-DNA or ds-RNA, which also agreed well with the literature.^48,49^ The ct-DNA was additionally sonicated and filtered through a 0.45 mm filter to obtain mostly short (*ca.* 100 base pairs) rod-like B-helical DNA fragments. Polynucleotide concentration was determined spectroscopically as the concentration of phosphates (corresponds to *c*(nucleobase)).^52^

### General synthetic procedure for coupling reactions

The suspension of a 4-(1-pyrenyl)butyric acid (0.1 mmol) in dry CH_3_CN (2 mL) was activated by means of HOBt (0.1 mmol) and HBTU (0.1 mmol), in the presence of bis-amino calix(4)arene (0.1 mmol) and Et_3_N (0.2 mmol) in an argon atmosphere. The reaction mixture was stirred at room temperature overnight. The solvent was removed under reduced pressure and the crude material was purified by preparative chromatography (CH_2_Cl_2_/MeOH 9 : 1) to obtain the desired product (Scheme 2). All characterization data of derivatives 2 and 3 (^1^H and ^13^C NMR spectra, HRMS spectra) are given in ESI (Fig. S1–S6†).

#### 5-Amino-11,23-di(*tert*-butyl)-17-[4-(pyren-1-yl)butanamido]-26,28-dimethoxy-25,27-bis[(1-methyl-1*H*-imidazole-2-yl)methoxy]calix[4]arene (2)

Compound 2 was prepared from 1 and 4-(1-pyrenyl)butyric acid by applying general procedure in 40% yield. ^1^H NMR (600 MHz, CD_3_CN) *δ* 8.41 (d, *J* = 9.2 Hz, 1H, Py), 8.24 (t, *J* = 6.7 Hz, 2H, Py), 8.18 (d, *J* = 4.0 Hz, 1H, Py), 8.17 (d, *J* = 2.4 Hz, 1H, Py), 8.13 (bs, 1H, Ar–NH–), 8.09 (d, *J* = 2.6 Hz, 2H, Py), 8.04 (t, *J* = 7.6 Hz, 1H, Py), 7.92 (d, *J* = 7.8 Hz, 1H, Py), 7.56 (s, 2H, H_Ar–NH–CO_), 7.33 (d, *J* = 2.4 Hz, 2H, H_Ar–*t*Bu_), 7.29 (d, *J* = 2.4 Hz, 2H, H_Ar–*t*Bu_), 7.25 (d, *J* = 1.3 Hz, 2H, H_Im_), 7.18 (d, *J* = 1.1 Hz, 2H, H_Im_), 6.55 (s, 2H, H_Ar–NH_2__), 4.98 (s, 4H, 2 × OCH_2_Im), 4.26 (d, *J* = 12.3 Hz, 2H, ArCH_2_Ar), 4.17 (d, *J* = 12.3 Hz, 2H, ArCH_2_Ar), 3.65 (s, 2H, ArCH_2_Ar), 3.57 (s, 6H, 2 × NCH_3_), 3.56 (s, 2H, ArCH_2_Ar), 3.46 (d, *J* = 12.4 Hz, 2H, –CH_2_–(Py)), 3.40–3.35 (m, 2H, –CH_2_–(Py)), 3.31 (d, *J* = 12.3 Hz, 2H,–CH_2_–(Py)), 2.73 (s, 6H, 2 ×OCH_3_), 2.38 (t, *J* = 7.1 Hz, 2H, NH_2_), 1.21 (s, 18H, ^*t*^Bu). ^13^C NMR (151 MHz, CD_3_CN) *δ* 172.03, 151.10, 150.43, 149.71, 146.45, 146.33, 145.33, 137.77, 137.58, 136.91, 136.84, 135.90, 135.39, 132.42, 131.95, 130.83, 129.66, 128.73, 128.51, 128.28, 128.21, 127.58, 127.57, 127.27, 127.12, 125.93, 125.92, 125.79, 125.77, 125.66, 124.73, 123.16, 120.78, 115.56, 71.93, 64.32, 64.13, 38.86, 37.12, 35.15, 33.30, 32.97, 31.50, 30.41, 30.39, 28.36. HRMS (ESI^+^, MeOH) *m*/*z*: calcd for C_68_H_73_N_6_O_5_ ([M + H]^+^) 1053.5642, found 1053.5631.

#### 5,17-Di[4-(pyren-1-yl)butanamido]-11,23-di(*tert*-butyl)-26,28-dimethoxy-25,27-bis[(1-methyl-1*H*-imidazole-2-yl)methoxy]calix[4]arene (3)

Compound 3 was prepared from 1 and 4-(1-pyrenyl)butyric acid by applying general procedure. Double moles of each component (HOBT/HBTU, Et_3_N) compared to limiting reactant 1 were used. Additionally, 2.5 eq. of 4-(1-pyrenyl)butyric acid (0.5 eq. excess compared to 1) was employed in the reaction. After purification, compound 3 was obtained in 40% yield. ^1^H NMR (600 MHz, CD_3_CN) *δ* 8.36 (d, *J* = 9.2 Hz, 2H, Py), 8.16 (d, *J* = 7.6 Hz, 4H, Py), 8.13 (bs, 2H, 2 × –NH–), 8.11 (d, *J* = 9.2 Hz, 2H, Py), 8.08 (d, *J* = 7.7 Hz, 2H, Py), 7.96–7.99 (m, 6H, Py), 7.87 (d, *J* = 7.7 Hz, 2H, Py), 7.55 (s, 4H, H_Ar–NH–CO_), 7.33 (s, 4H, H_Ar–*t*Bu_), 7.26 (s, 2H, H_Im_), 7.19 (s, 2H, H_Im_), 5.00 (s, 4H, 2 × OCH_2_Im), 4.26 (d, *J* = 12.5 Hz, 4H, ArCH_2_Ar), 3.65 (s, 4H, ArCH_2_Ar), 3.58 (s, 6H, 2 × NCH_3_), 3.46 (d, *J* = 12.5 Hz, 4H, –CH_2_–(Py)), 3.35–3.32 (m, 4H, –CH_2_–(Py)), 2.73 (s, 6H, 2 × OCH_3_), 2.36 (t, *J* = 6.9 Hz, 4H, –CH_2_–(Py)), 1.17 (s, 18H, ^*t*^Bu). ^13^C NMR (151 MHz, CD_3_CN) *δ* 172.05, 159.94, 150.95, 150.73, 145.46, 145.32, 136.92, 132.35, 130.76, 128.70, 128.42, 128.28, 128.17, 127.54, 127.49, 127.05, 125.88, 125.85, 125.73, 124.68, 123.19, 122.62, 120.78, 64.75, 55.29, 38.86, 37.07, 33.26, 32.99, 31.49, 30.38, 28.33. HRMS (ESI^+^, MeOH) *m*/*z*: calcd for C_88_H_87_N_6_O_6_ ([M + H]^+^) 1323.6687, found 1323.6683.

### Photophysical properties

TC-SPC measurements were performed on an Edinburgh FS5 spectrometer equipped with a pulsed LED at 340 nm. Fluorescence signals at 400 and 475 nm were monitored over 1023 channels with a time increment of 488 ps per channel. The decays were collected until they reached counts of range 3000 in the peak channel. A suspension of silica gel in H_2_O was used as a scattering solution to obtain instrument response function (IRF). Absorbances at 340 nm were 0.07–0.09. Prior to the measurements, the solutions were purged with a stream of argon for 20 min. The measurement was performed at rt (25 °C). Decays of fluorescence were fit to a sum of exponentials according to eqn (1):

1 *R*(*t*) = *A* + *B*_1_e^−*t*/*τ*_1_^ + *B*_2_e^−*t*/*τ*_2_^ + *B*_3_e^−*t*/*τ*_2_^

Using software implemented with the instrument; absolute quantum yields were determined by the Integrating sphere SC-30 of the Edinburgh FS5 spectrometer in the quartz cuvette of 10 mm path length, to avoid the scattering of incident light at the liquid–air interface, testing solutions with a 2 mL volume were used.

### Study of interactions with nucleotides and ds-DNA/RNA

All measurements of interactions with nucleotides and ds-DNA/RNA were performed in aqueous buffer solution (pH = 7.0, sodium cacodylate buffer, *I* = 0.05 M). The UV-Vis spectra were recorded on a Varian Cary 100 Bio spectrometer, fluorescence spectra were recorded on a Varian Cary Eclipse fluorimeter, and CD spectra were recorded on JASCO J815 spectropolarimeter at 25.0 °C, equipped with a thermostat, using appropriate quartz cuvettes (Hellma Suprasil QX, path length: 1 cm). The absorption spectral changes of 2 and 3 (*c* = 2 × 10^−6^ to 2 × 10^−5^ M, *V*_0_ = 1 mL) were recorded upon stepwise addition of nucleotide and DNA/RNA stock solutions (*c* = 6.7 × 10^−6^ to 4 × 10^−4^ M) into the cuvette. The absorbances at *λ*_exc_ = 350 nm were sampled at 1 nm intervals, with an integration time of 10 s. Fluorometric titrations at *λ*_exc_ = 350 nm were performed by adding aliquots of nucleotides and polynucleotide stock solutions (*c* = 5 × 10^−6^ to 8 × 10^−3^ M) to the calixarene solution (*c* = 5 × 10^−7^ to 2 × 10^−6^ M, *V*_0_ = 1.5 mL). The obtained UV and fluorometric data were corrected for the dilution and used for calculation of binding constants by fitting to 1 : 1 stoichiometry of complex formed.

Thermal melting curves for ds-DNA, ds-RNA, and their complexes with studied compounds were determined as previously described by monitoring the absorption change at 260 nm as a function of temperature.^50^ The absorbance of the ligands was subtracted from every curve, and the absorbance scale was normalized. *T*_m_ values are the midpoints of the transition curves determined from the maximum of the first derivative and checked graphically by the tangent method. The Δ*T*_m_ values were calculated subtracting *T*_m_ of the free nucleic acid from *T*_m_ of the complex. Every Δ*T*_m_ value here reported was the average of at least two measurements. The error in Δ*T*_m_ is ±0.5 °C.

CD experiments were performed by adding portions of the compound stock solution (*c* = 10^−3^ to 5 × 10^−3^ M) into the polynucleotide solution (*c* = 3 × 10^−5^ M, *V*_0_ = 2 mL). The spectra were recorded as an average of the accumulations with a scanning speed of 200 nm min^−1^ and a buffer background was subtracted from each spectrum.

All results were processed by using the OriginPro 7.0 program.

### Computational methods

Molecular dynamics simulations (MD) were done in Amber2020 program package.^53^ Mono- and bis-pyrene derivatives were described using GAFF2 (Generalized Force Field 2) force field parameters that are suggested for organic molecules like ligands.^54^ The system was simulated in TIP3P water cubical box (dimension 11.0 Å),^55^ with periodic boundary conditions, consisting of few thousand of water molecules treated explicitly. When simulating the calixarenes in mono-cationic form the chlorine anion was added to neutralize the system.

The MD simulations were performed in 4 parts: minimization, heating, equilibration and the final dynamics. Geometry optimization was done with steepest descent and conjugated gradient method. The system was heated from 100 to 300 K using Langevin thermostat at constant volume (*NVT* ensemble).^56^ Equilibration step followed at constant temperature and pressure: *T* = 300 K, *p* = 1 atm (*NpT* ensemble). The pressure was kept at 1 bar on average using Berendsen thermostat^57^ and the temperature (300 K) was regulated with Langevin thermostat. The molecular dynamics simulation was performed *T* = 300 K for 10 ns with a timestep 0.1 fs. The cut-off radius for nonbonded van der Waals and short-range Coulomb interactions was 9 Å. Long-range Coulomb interactions were treated by the Ewald method, as implemented in the particle mesh Ewald (PME) procedure.^58^ Figure of molecular structure was created using Pymol software.^59^

## Author contributions

I. N.-F., D. P. S. and A. V. performed synthetic procedures. I. N.-F. performed spectroscopic measurements. B. C. and O. R. designed studied compounds and contributed in writing of the paper; I. N.-F. performed computational analysis, relative quantum yield determination, fluorescence lifetime measurements and contributed to writing of the paper; A. V. assisted in design of the research, contributed to writing of the paper and procured financial support, while I. P. designed the research concept, analysed and supervised the experimental results. D. P. S. wrote the paper. All authors have critically read and agreed to the published version of the manuscript.

## Conflicts of interest

There are no conflicts of interest to declare.

## Supplementary Material

RA-013-D3RA05696A-s001
