# Supplementary material for: Novel pyrene-calix[4]arene derivatives as highly sensitive sensors for nucleotides, DNA and RNA
Source: RSC Adv. 2023 Sep 13;13(39):27423–33. doi: 10.1039/d3ra05696a (PMC10498358; doi:10.1039/d3ra05696a)
Supplement: RA-013-D3RA05696A-s001 [file RA-013-D3RA05696A-s001.pdf]

Electronic Supplementary Material (ESI) for RSC Advances.
This journal is © The Royal Society of Chemistry 2023

Electronic Supplementary Information

Novel Pyrene-Calix[4]arene Derivatives as Highly Sensitive Sensors for Nucleotides, DNA and RNA

Ivana Nikšić-Franjić,[a] Benoit Collason,[b] Olivia Reinaud,[b] Aleksandar Višnjevac,[a,*] Ivo Piantanida[c] and Dijana Pavlović Saftić[c,*]

[a] *Division of Physical Chemistry, Laboratory for chemical and biological crystallography, Ruđer Bošković Institute, Bijenička cesta 54, 10000 Zagreb, Croatia*
[b] *Université de Paris - Laboratoire de Chimie et Biochimie Pharmacologiques et Toxicologiques, CNRS UMR 8601, 45 rue des Saints Pères, 75006 Paris, France*
[c] *Division of Organic Chemistry and Biochemistry, Laboratory for Biomolecular Interactions and Spectroscopy, Ruđer Bošković Institute, Bijenička cesta 54, 10000 Zagreb, Croatia*

*Corresponding authors:

Dijana Pavlović-Saftić, e-mail; dijana.pavlovic.saftic@irb.hr
Aleksandar Višnjevac, e-mail: aleksandar.visnjevac@irb.hr

Contents

1. NMR and HRMS spectra of 2 and 3 1

3. Interactions with DNA and RNA 11

3.1. Spectrophotometric titrations with mono- and polynucleotides 12

3.2. Circular dichroism (CD) experiments 30

3.3. Thermal denaturation experiments 34

1. NMR and HRMS spectra of 2 and 3

General procedures

Solvents were distilled from appropriate drying agents shortly before use. TLC was carried out on DC-plastikfolien Kieselgel 60 F_{254} and preparative thick layer (2 mm) chromatography was done on Merck 60 F_{254}. ^{1}H and ^{13}C NMR spectra were recorded in CD_3CN on Bruker AV 600 MHz spectrometers. Chemical shifts (δ) are expressed in ppm, and *J* values in Hz. Signal multiplicities are denoted as s (singlet), d (doublet), t (triplet), q (quartet) and m (multiplet). High resolution mass spectra (HRMS) were obtained using a MALDI-TOF/TOF mass spectrometer 4800 Plus MALDI TOF/TOF analyzer (Applied Biosystems Inc., Foster City, CA, USA).

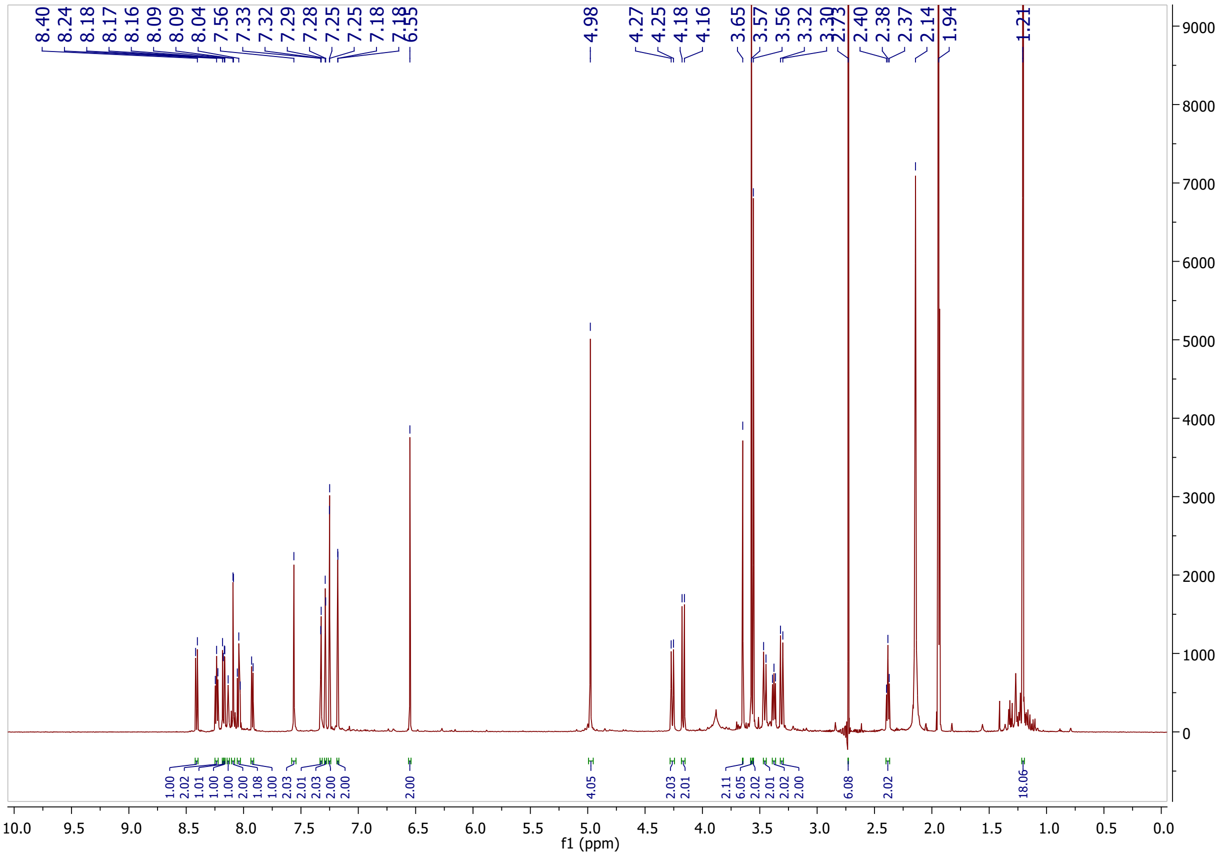

Figure S1. ^{1}H NMR (600 MHz) spectrum of compound **2** recorded in CD_3CN.

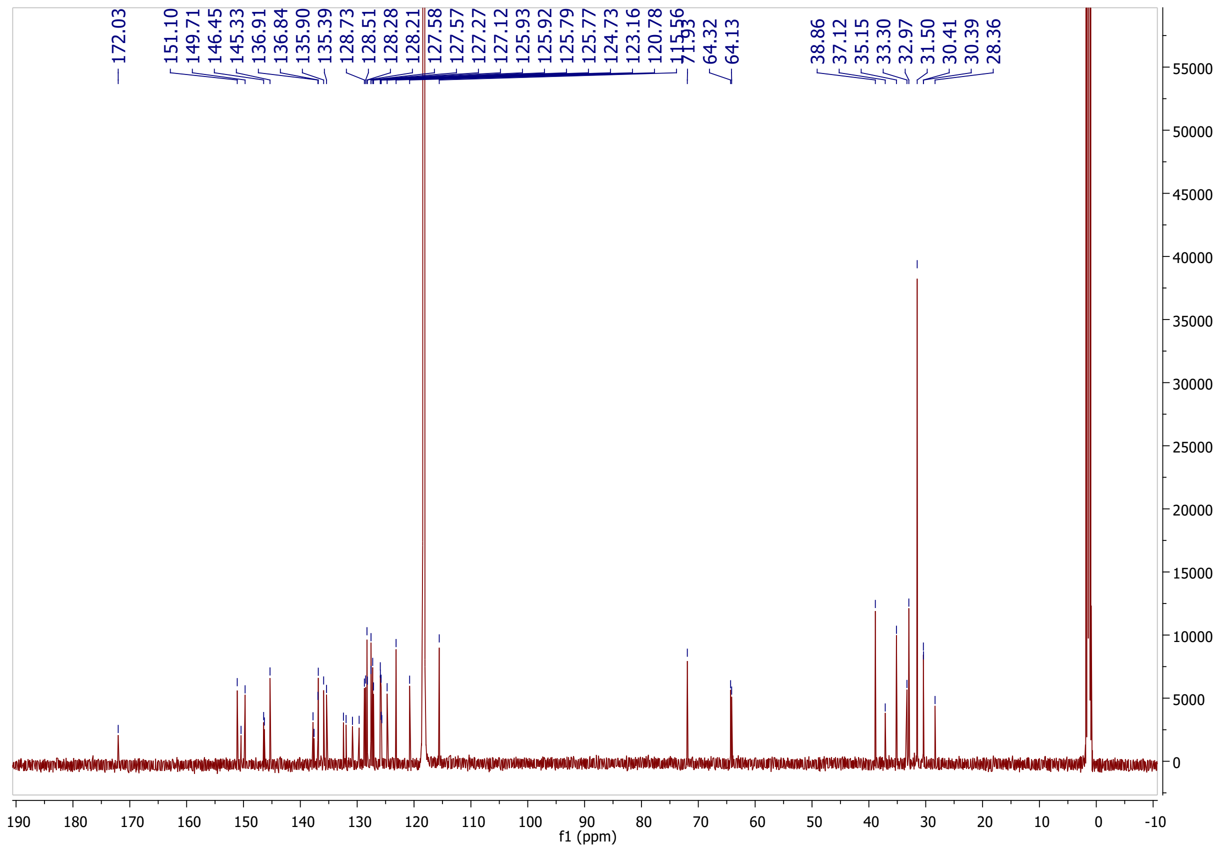

Figure S2. ^{13}C NMR (151 MHz) spectrum of compound **2** recorded in CD_3CN.

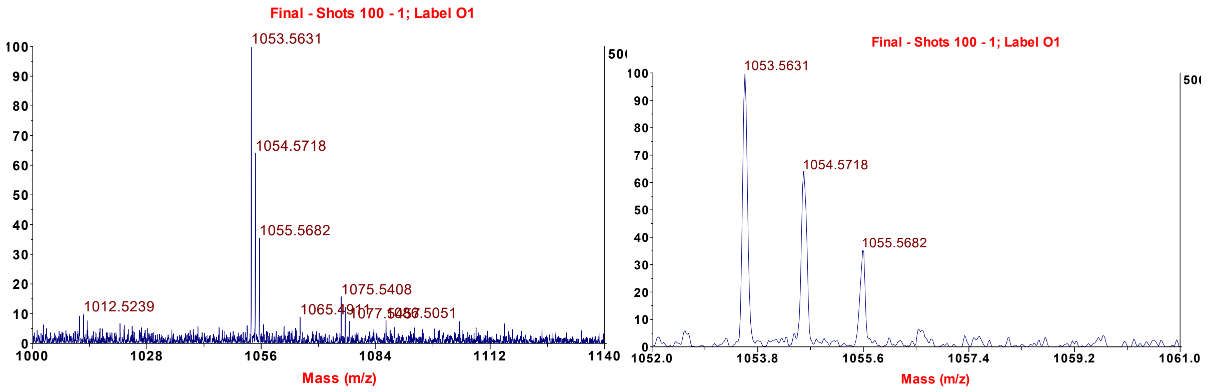

Figure S3. MALDI TOF/TOF mass spectrum of compound **2** in the range *m/z* 1000 – 1140 (left), and magnification of the spectrum from *m/z* 1052 to *m/z* 1061 (right).

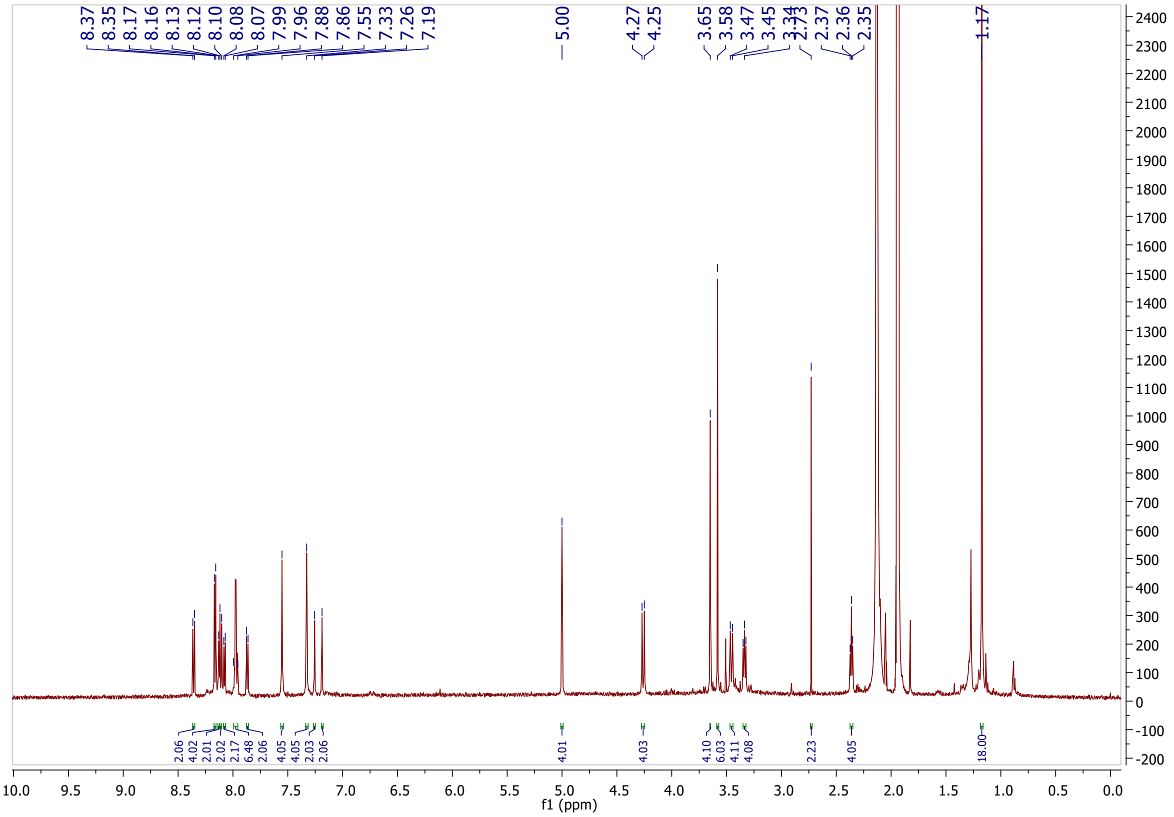

Figure S4. ^{1}H NMR (600 MHz) spectrum of compound **3** recorded in CD_3CN.

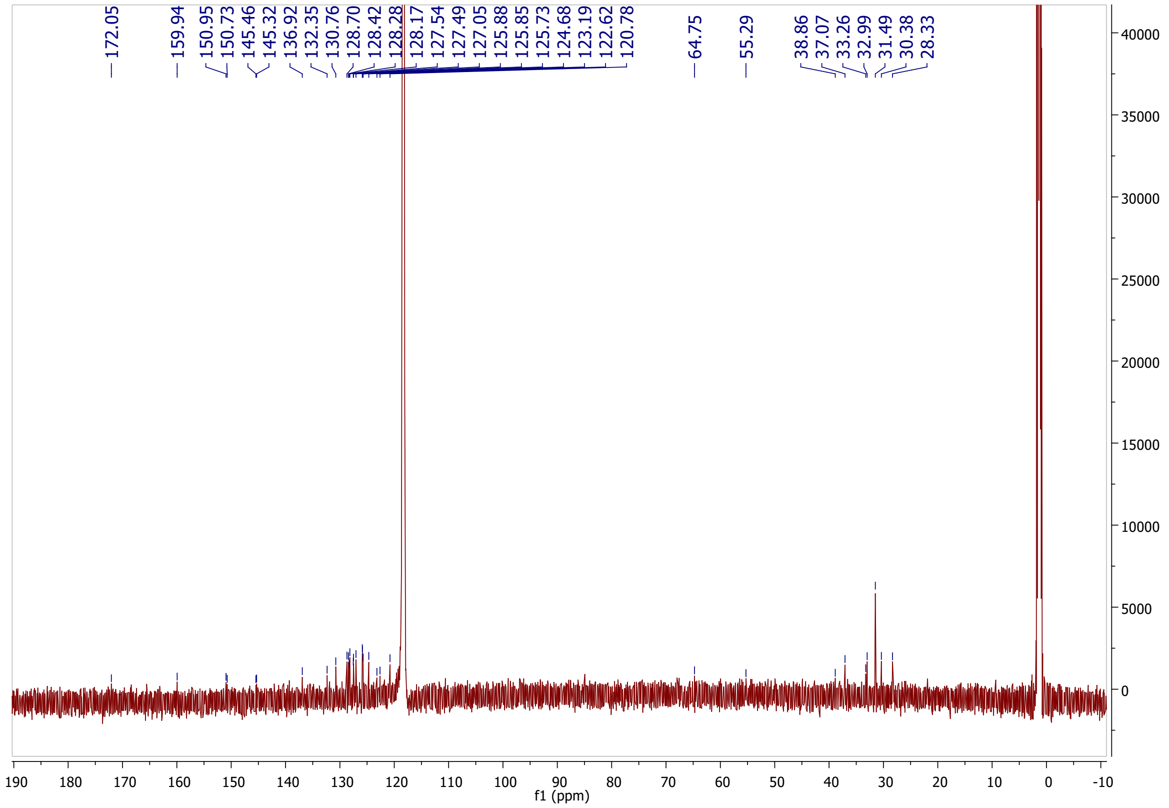

Figure S5. ^{13}C NMR (151 MHz) spectrum of compound **3** recorded in CD_3CN.

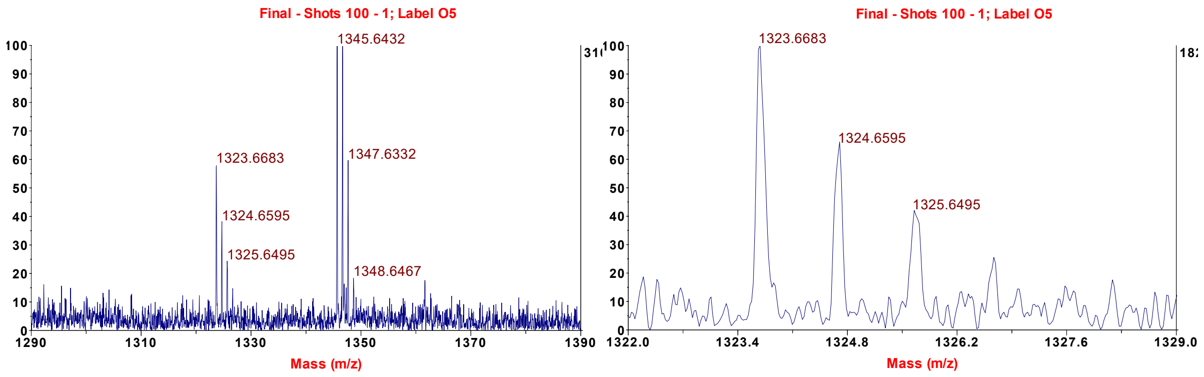

Figure S6. MALDI TOF/TOF mass spectrum of compound **3** in the range *m/z* 1290 – 1390 (left), and magnification of the spectrum from *m/z* 1322 to *m/z* 1329 (right).

2. Spectrofluorimetric and photophysical characterization

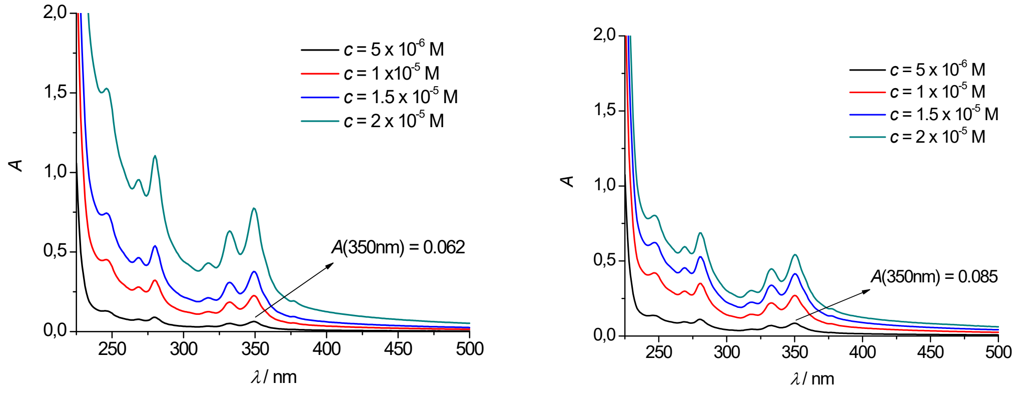

a)

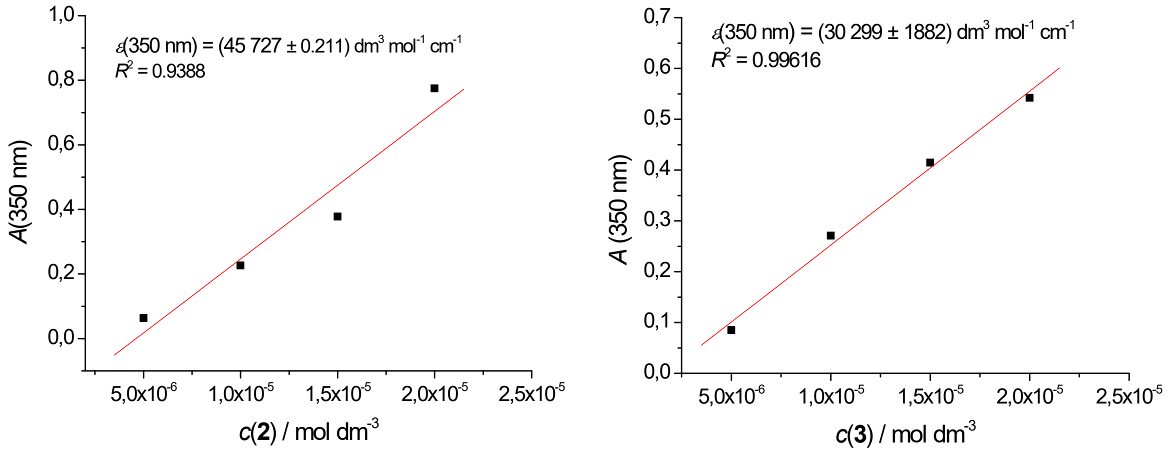

b)

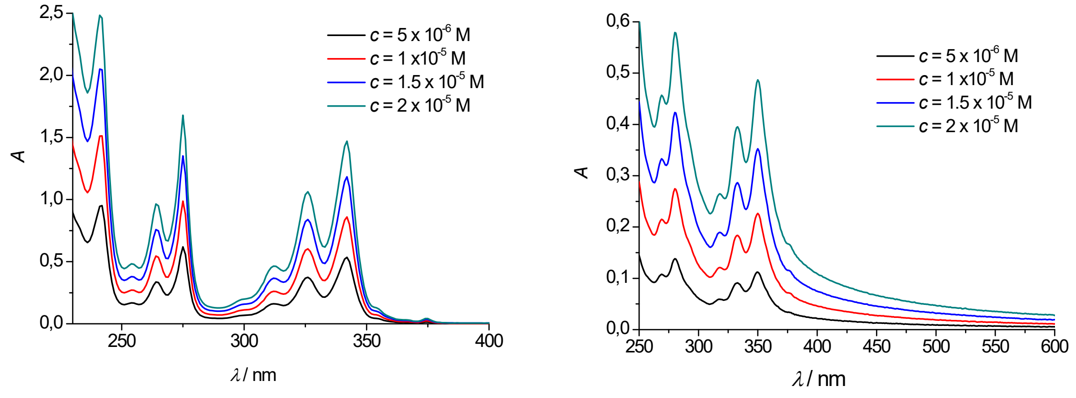

c)

Figure S7. (a) UV/Vis spectra of studied conjugates **2** and **3** in water (0.1% DMSO); (b) absorbance dependence on *c*(**2** or **3**) ($c = 5 \times 10^{-6} - 2 \times 10^{-5}$ M); (c) UV/Vis spectra of referent compounds 1-pyrenebutyric acid (**A**) and **B** in buffered solution pH 7, $I = 0.05$ M.

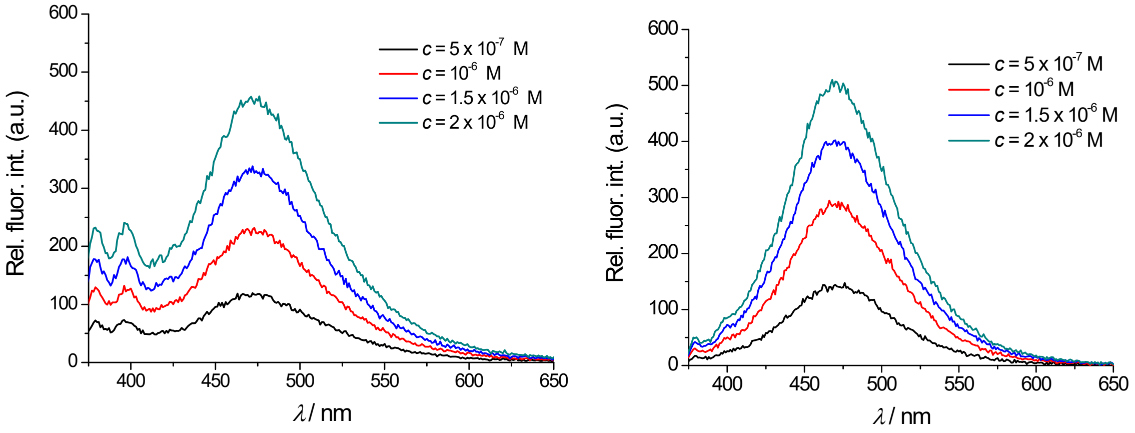

a) b)

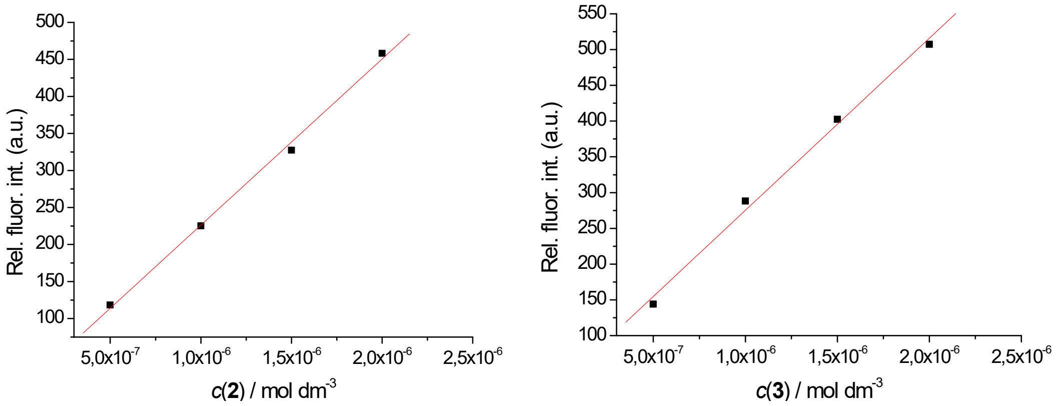

c)

Figure S8. Fluorescence spectra of: a) **2** and b) **3**; c) relative fluorescence dependence on *c*(**2** or **3**) ($c = 5 \times 10^{-7} - 2 \times 10^{-6}$ M) at $\lambda_{em} = 475$ nm. Done in water, $\lambda_{exc} = 350$ nm.

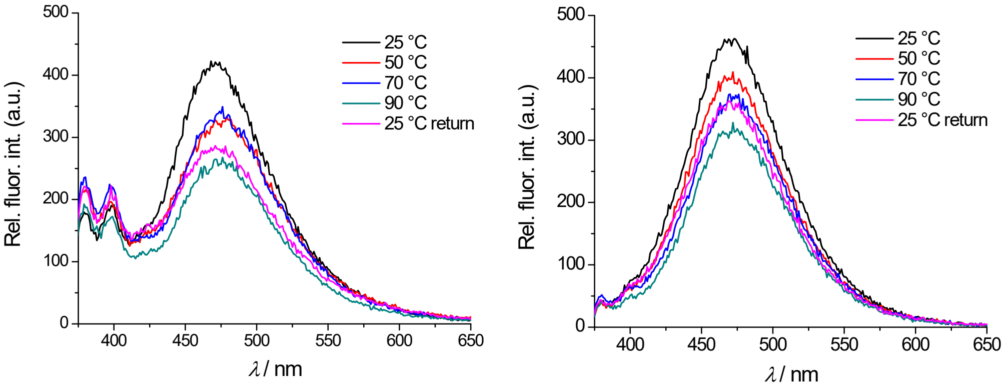

a) b)

Figure S9. Temperature dependence of: a) **2** and b) **3** at concentration $c = 2 \times 10^{-6}$ M. Done in water, $\lambda_{exc} = 350$ nm.

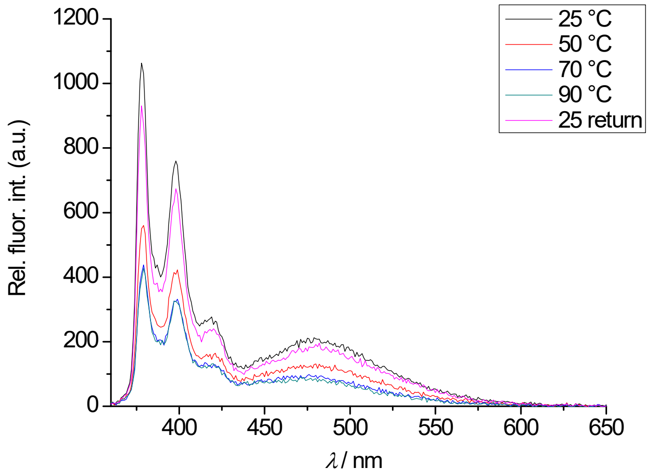

Figure S10. Temperature dependence of **3** at concentration $c = 2 \times 10^{-6}$ M. Done in DMSO, $l = 1$ cm, $\lambda_{exc} = 350$ nm.

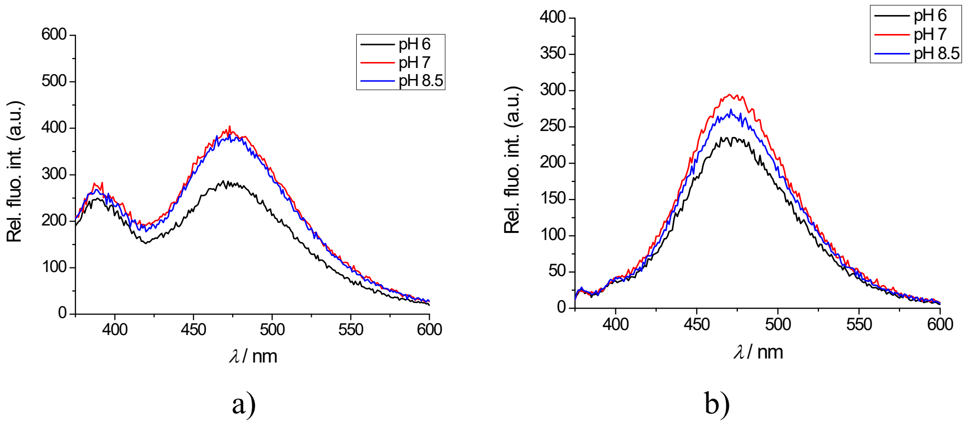

Figure S11. The dependence of the fluorescence spectra of compounds **2** (a) and **3** (b) ($c = 1 \times 10^{-6}$ M) on pH. Done in phosphate buffer adjusted to various pH values by buffer preparation protocol.

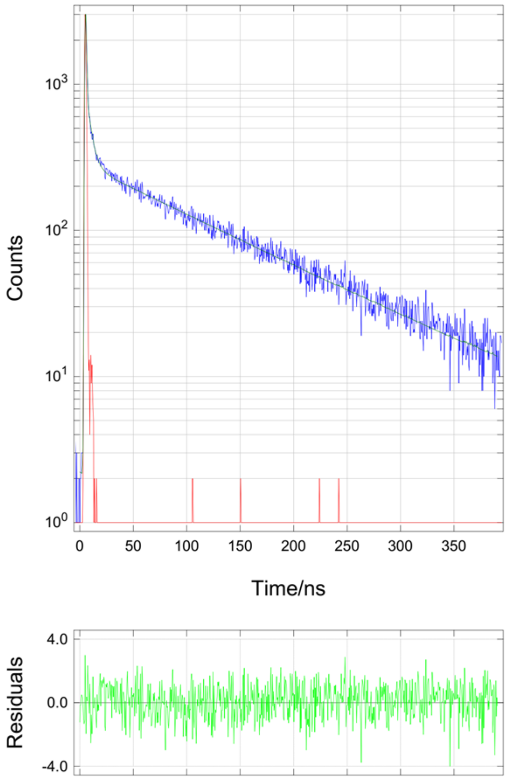

a) 400 nm, water

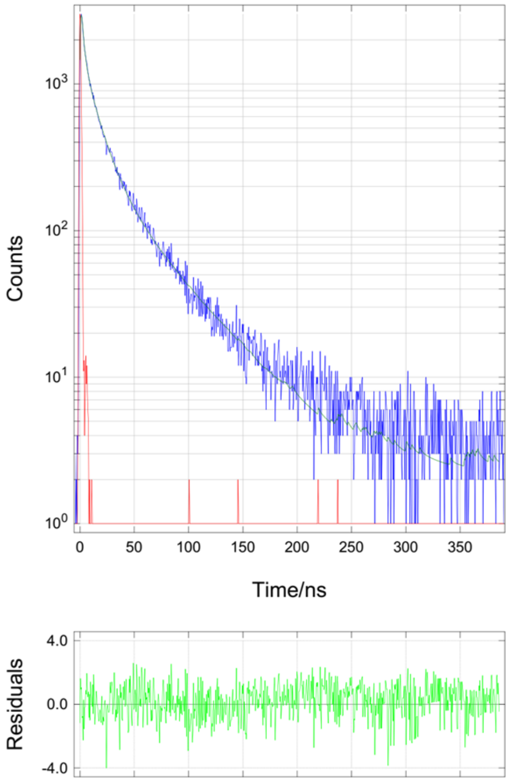

b) 475 nm, water

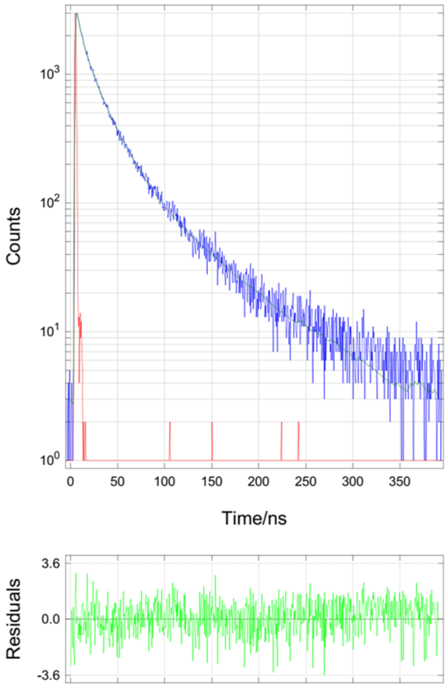

c) 400 nm, DMSO

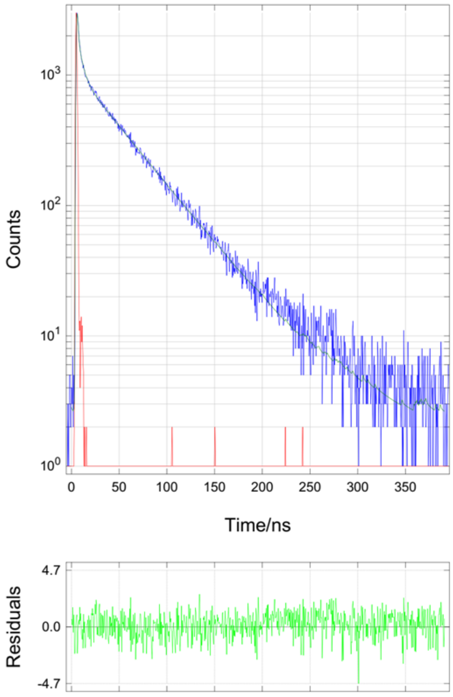

d) 475 nm, DMSO

Figure S12. Time resolved fluorescence decay for **2** (c = 10^{-6} M) in water and DMSO at emission wavelengths: 400 and 475 nm. Blue: **2** decay, red: IRF (instrument time response time scan), green: weighted residuals time scan.

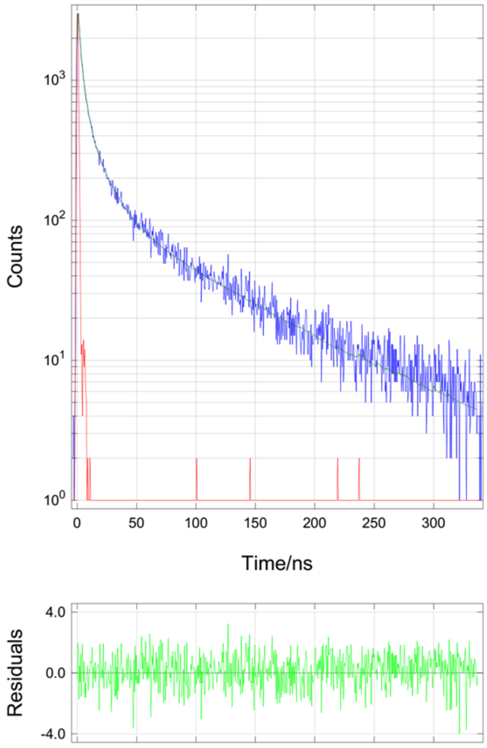

a) 400 nm, water

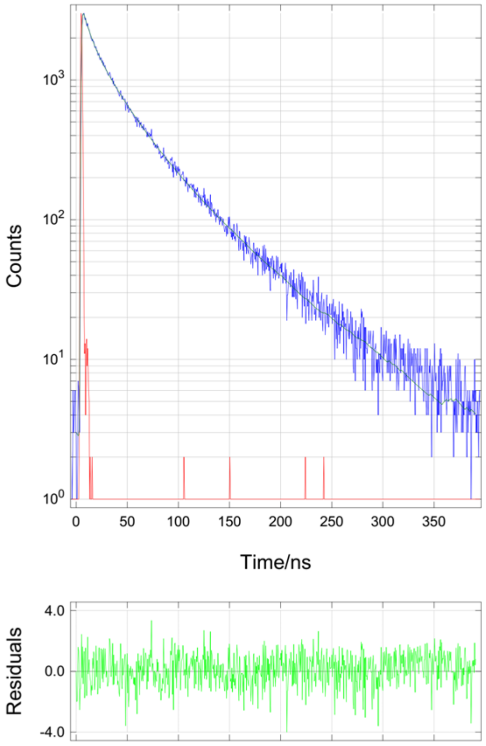

b) 475 nm, water

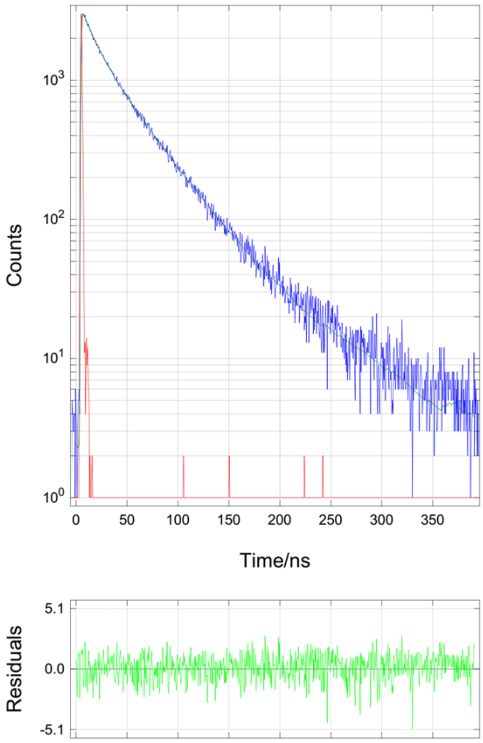

c) 400 nm, DMSO

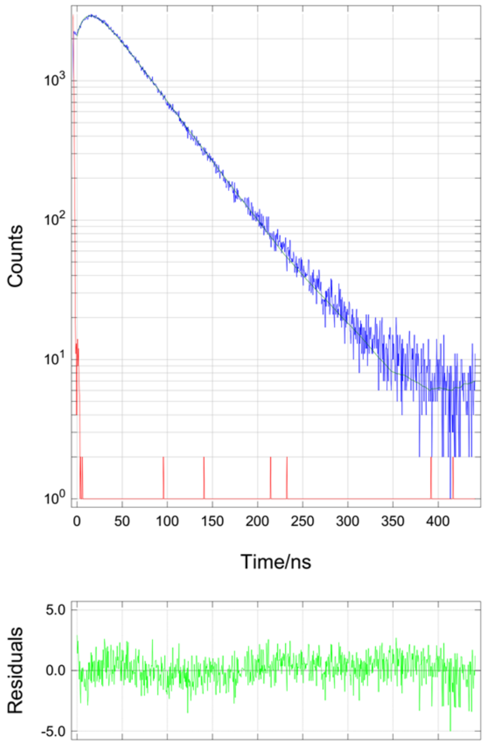

d) 475 nm, DMSO

Figure S13. Time resolved fluorescence decay for **3** (c = 10^{-6} M) in water and DMSO at emission wavelengths: 400 and 475 nm. Blue: **3** decay, red: IRF (instrument time response time scan), green: weighted residuals time scan.

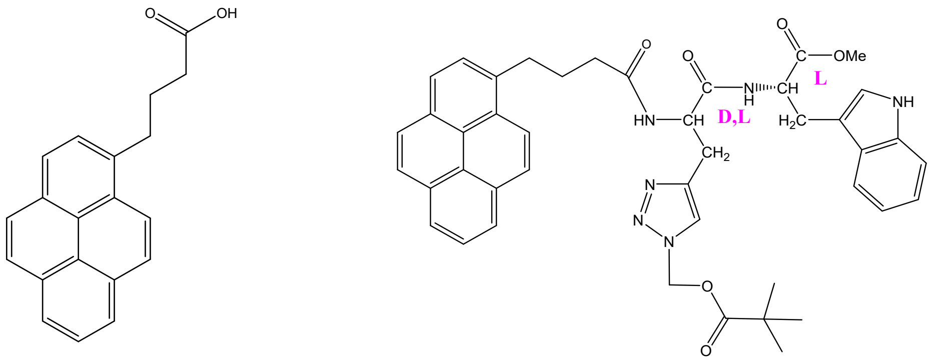

A = 1-pyrenebutyric acid **B** = PyrL-Ala(Triazole-R)-Trp

Figure S14. Referent compounds **A** and **B** previously published.[1]

[1] I. Krošl, M. Košćak, K. Ribičić, B. Žinić, D. Majhen, K. Božinović and I. Piantanida, Impact of the Histidine-Triazole and Tryptophan-Pyrene Exchange in the WHW Peptide: Cu(II) Binding, DNA/RNA Interactions and Bioactivity, *Int. J. Mol. Sci*. 2022, **23**, 7006.

3. Interactions with DNA and RNA

Table S1. Structural features of selected nucleic acids.[2,3]

Structure type	Groove width [Å]		Groove depth [Å]	
	major	minor	major	minor
**poly A- poly U (A-helix)**	3.8	10.9	13.5	2.8
**ct-DNA (B-helix)**	11.7	5.7	8.5	7.5
**$(dGdC)_n$ (B-helix)**	13.5	9.5	10.0	7.2
**$p(dAdT)_2$ (B-helix)**	11.2	6.3	-	-

[2] W. Saenger, *Principles of Nucleic Acid Structure*, Springer-Verlag, New York, 1983.
[3] C. R. Cantor, P. R. Schimmel, *Biophysical Chemistry*, 1980, **3**, 1109-1181.

3.1. Spectrophotometric titrations with mono- and polynucleotides

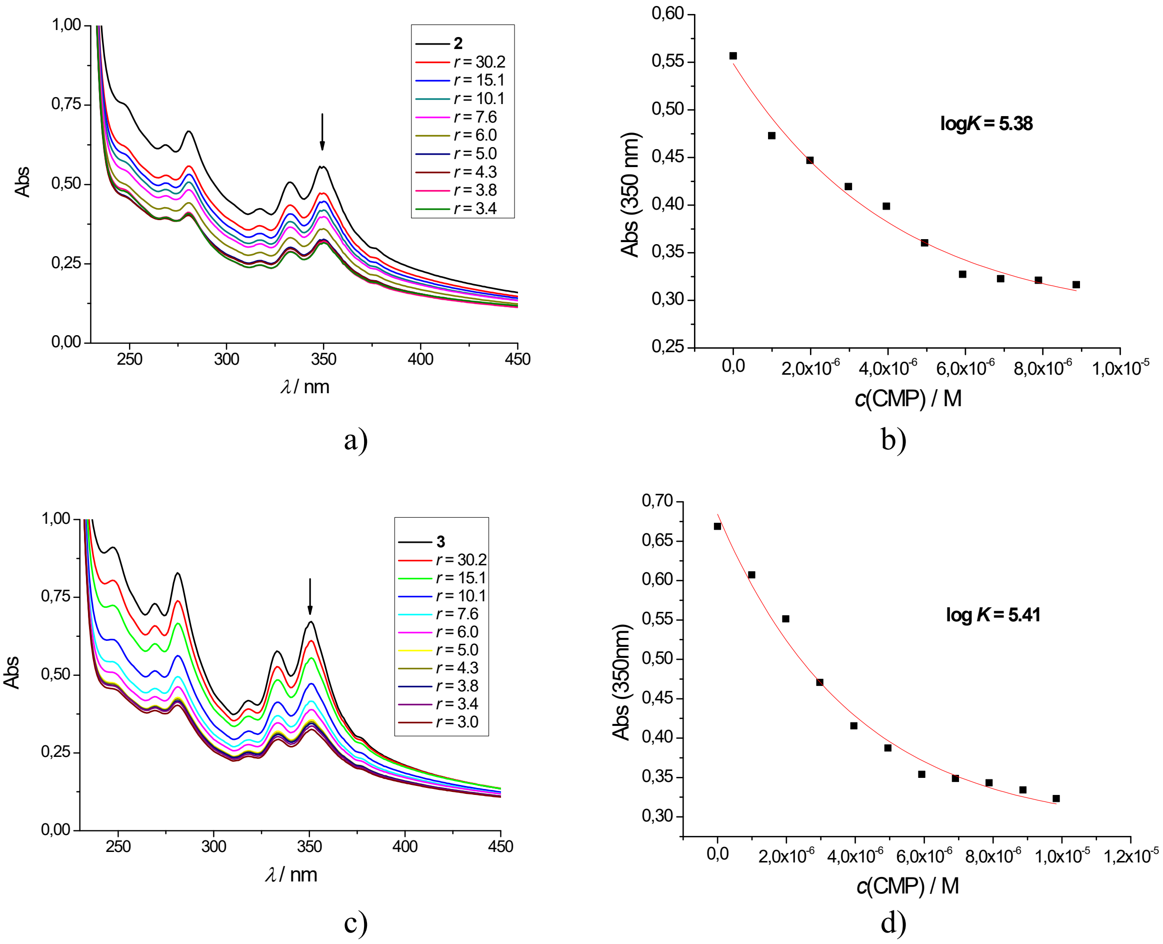

Figure S15. a) Spectrophotometric titration of **2** (c = 3 ×10^{-5} M) with CMP (c = 1 × 10^{-3} M) and b) dependence of absorption at λ_{max}= 350 nm on c(CMP); c) spectrophotometric titration of **3** (c = 3 ×10^{-5} M) with CMP (c = 1 × 10^{-3} M) and d) dependence of absorption at λ_{max}= 350 nm on c(CMP). Done in sodium cacodylate buffer (pH=7.0, I = 0.05 M); r = [compound] / [mononucleotide].

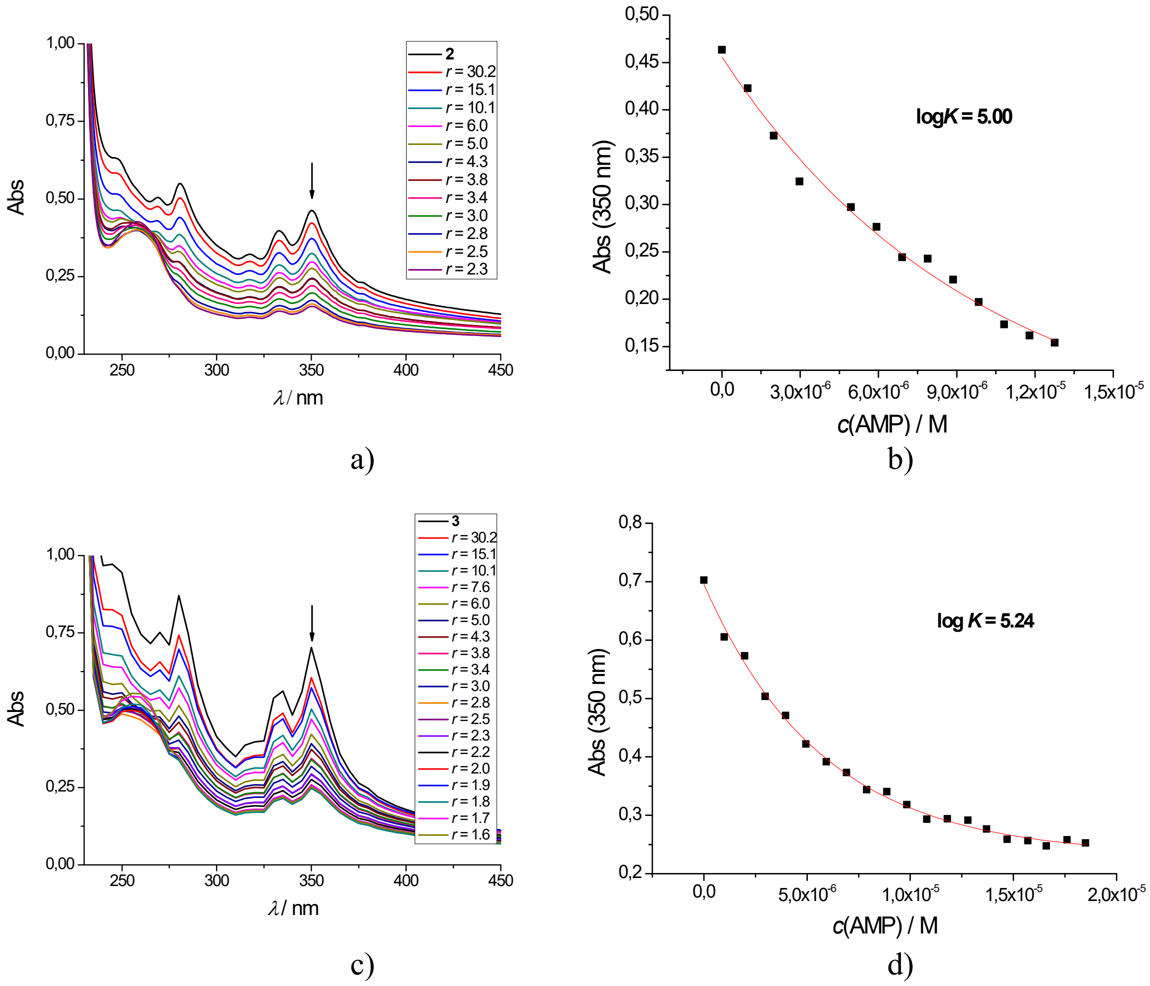

Figure S16. a) Spectrophotometric titration of **2** ($c = 3 \times 10^{-5}$ M) with AMP ($c = 1 \times 10^{-3}$ M) and b) dependence of absorption at λ_{max}= 350 nm on c(AMP); c) spectrophotometric titration of **3** ($c = 3 \times 10^{-5}$ M) with AMP ($c = 1 \times 10^{-3}$ M) and d) dependence of absorption at λ_{max}= 350 nm on c(AMP). Done in sodium cacodylate buffer (pH=7.0, I = 0.05 M); r = [compound] / [mononucleotide].

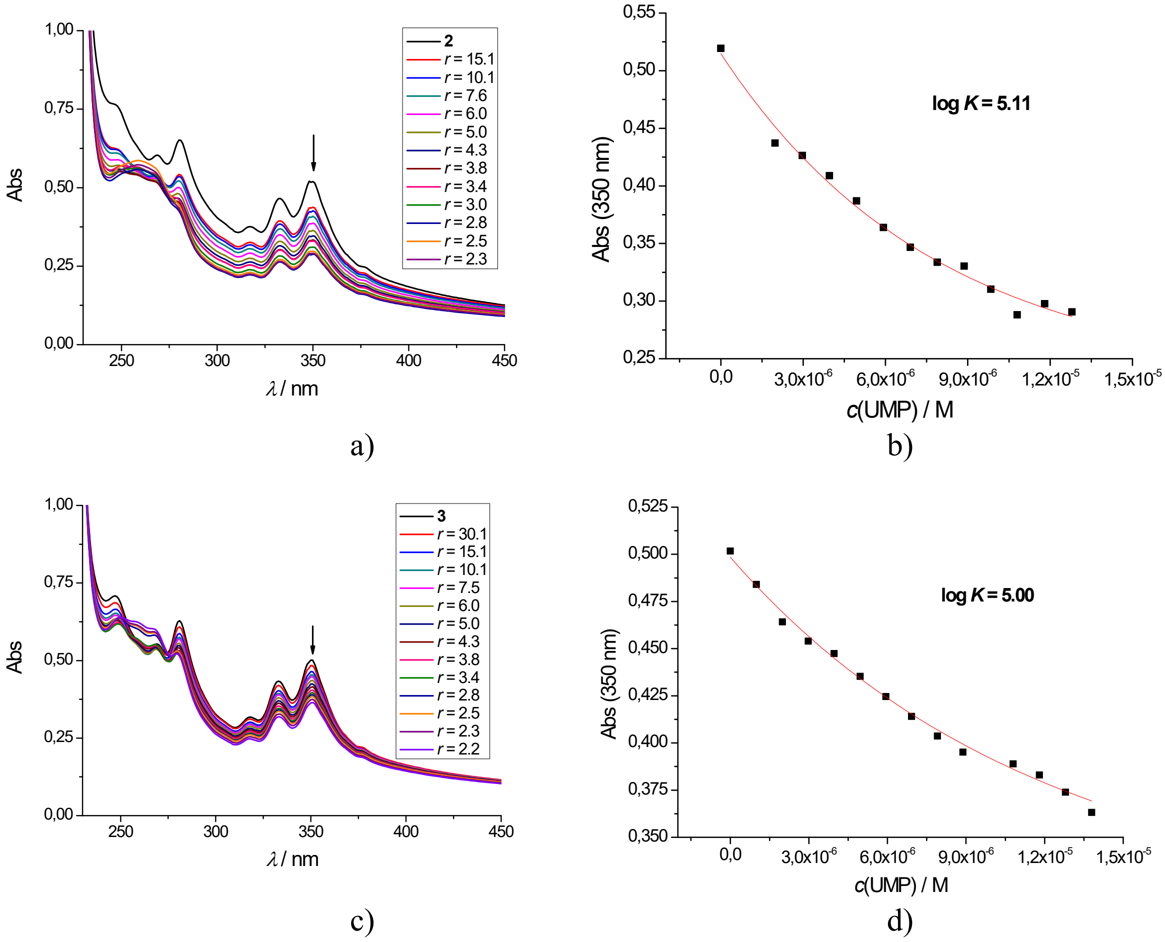

Figure S17. a) Spectrophotometric titration of **2** ($c = 3 \times 10^{-5}$ M) with UMP ($c = 1 \times 10^{-3}$ M) and b) dependence of absorption at λ_{max}= 350 nm on c(UMP); c) spectrophotometric titration of **3** ($c = 3 \times 10^{-5}$ M) with UMP ($c = 1 \times 10^{-3}$ M) and d) dependence of absorption at λ_{max}= 350 nm on c(UMP). Done in sodium cacodylate buffer (pH=7.0, I = 0.05 M); r = [compound] / [mononucleotide].

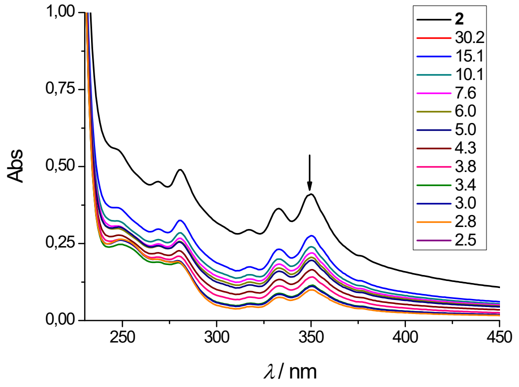

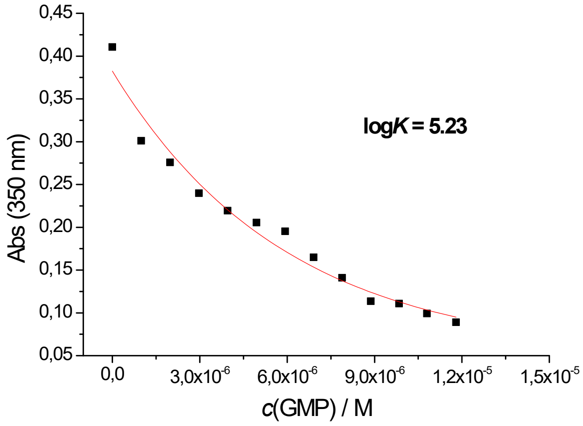

a) b)

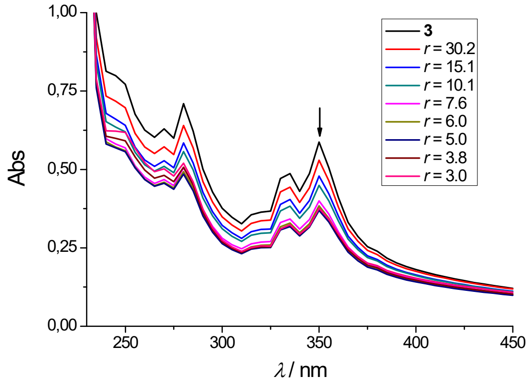

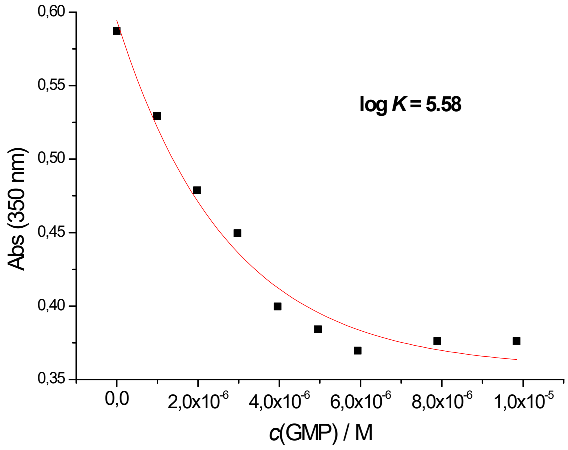

c) d)

Figure S18. a) Spectrophotometric titration of **2** (c = 3 $\times 10^{-5}$ M) with GMP (c = 1 $\times$ 10^{-3} M) and b) dependence of absorption at λ_{max}= 350 nm on c(GMP); c) spectrophotometric titration of **3** (c = 3 $\times 10^{-5}$ M) with GMP (c = 1 $\times$ 10^{-3} M) and d) dependence of absorption at λ_{max}= 350 nm on c(GMP). Done in sodium cacodylate buffer (pH=7.0, I = 0.05 M); r = [compound] / [mononucleotide].

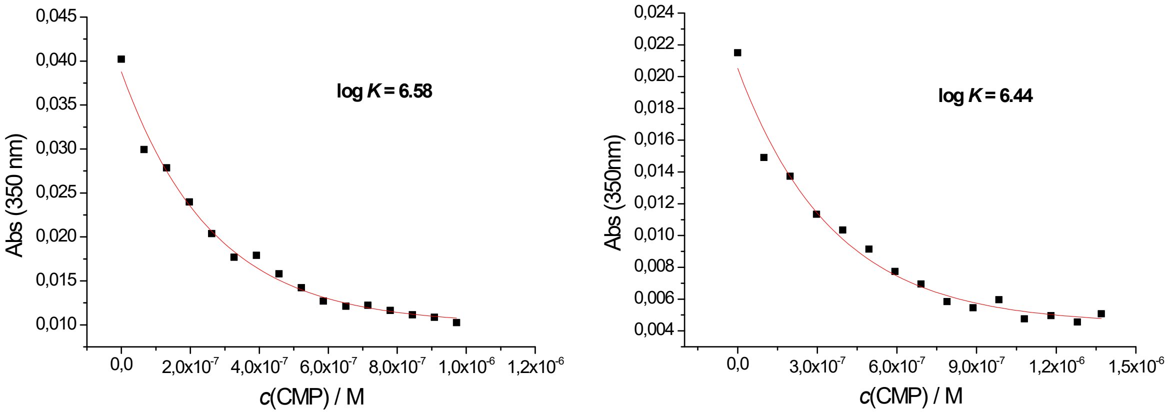

a) b)

Figure S19. a) Absorption dependence of **2** (c = 2 × 10^{-6} M) on concentration of CMP (c = 6.67 × 10^{-5} M) at λ_{max}= 350 nm on c(CMP); b) Absorption dependence of **3** (c = 3 × 10^{-6} M) on concentration of CMP (c = 1 × 10^{-4} M) at λ_{max}= 350 nm on c(CMP). Done in sodium cacodylate buffer (pH=7.0, I = 0.05 M).

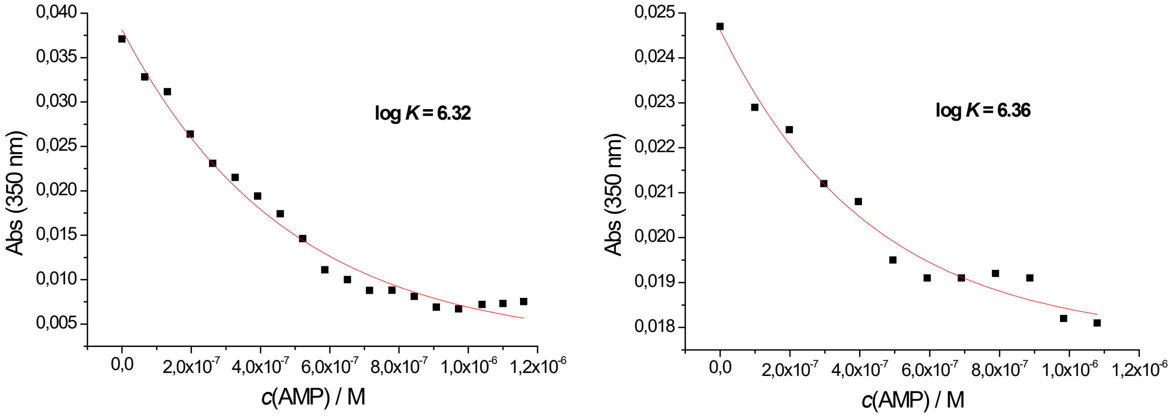

a) b)

Figure S20. a) Absorption dependence of **2** (c = 2 × 10^{-6} M) on concentration of AMP (c = 6.67 × 10^{-5} M) at λ_{max}= 350 nm on c(AMP); b) Absorption dependence of **3** (c = 3 × 10^{-6} M) on concentration of AMP (c = 1 × 10^{-4} M) at λ_{max}= 350 nm on c(AMP). Done in sodium cacodylate buffer (pH=7.0, I = 0.05 M).

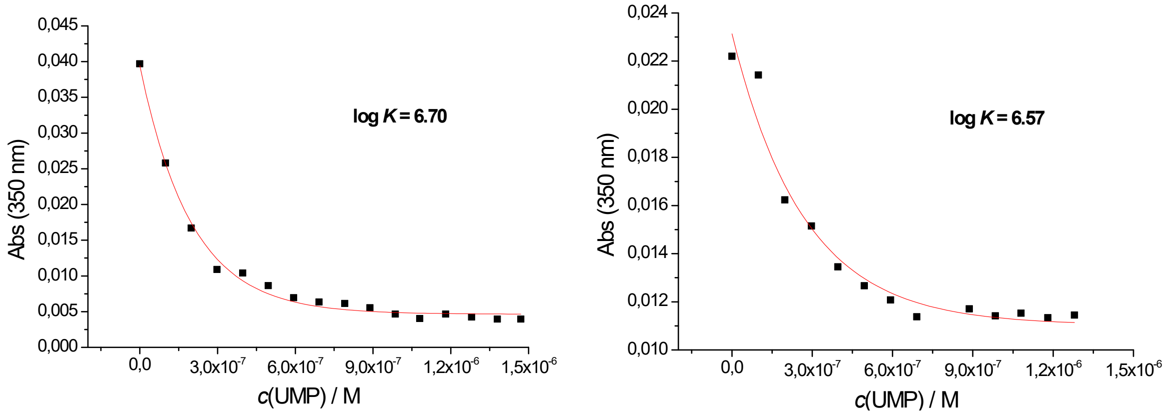

a) b)

Figure S21. a) Absorption dependence of **2** ($c = 2 \times 10^{-6}$ M) on concentration of UMP ($c = 1 \times 10^{-4}$ M) at λ_{max}= 350 nm on c(UMP); b) Absorption dependence of **3** ($c = 3 \times 10^{-6}$ M) on concentration of UMP ($c = 1 \times 10^{-4}$ M) at λ_{max}= 350 nm on c(UMP). Done in sodium cacodylate buffer (pH=7.0, I = 0.05 M).

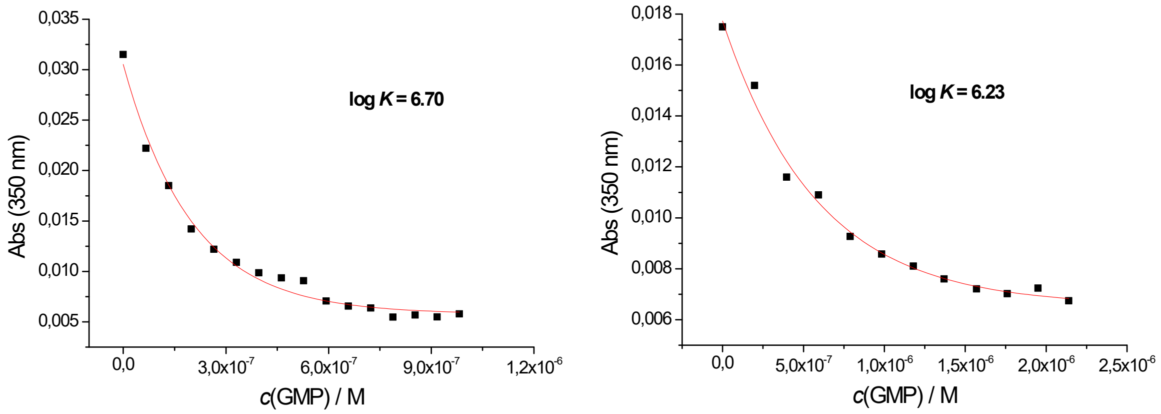

a) b)

Figure S22. a) Absorption dependence of **2** ($c = 2 \times 10^{-6}$ M) on concentration of GMP ($c = 6.67 \times 10^{-5}$ M) at λ_{max}= 350 nm on c(GMP); b) Absorption dependence of **3** ($c = 3 \times 10^{-6}$ M) on concentration of GMP ($c = 1 \times 10^{-4}$ M) at λ_{max}= 350 nm on c(GMP). Done in sodium cacodylate buffer (pH=7.0, I = 0.05 M).

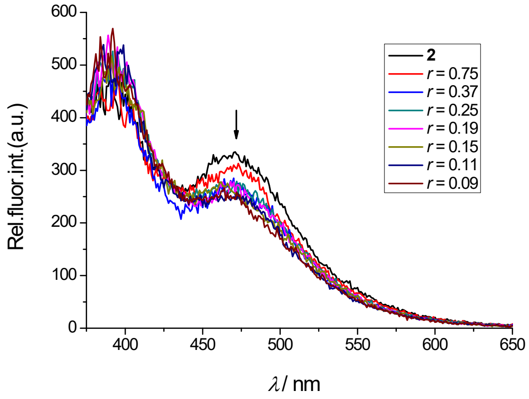

a)

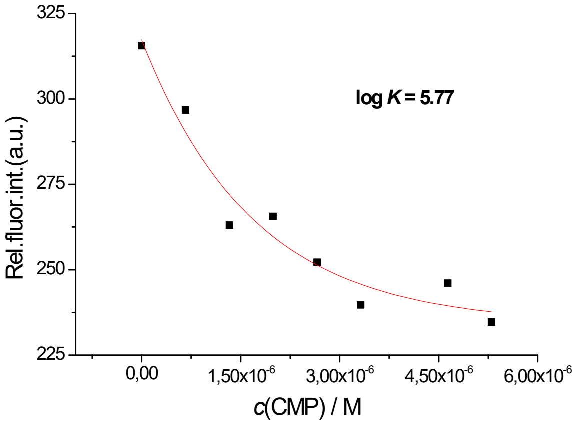

b)

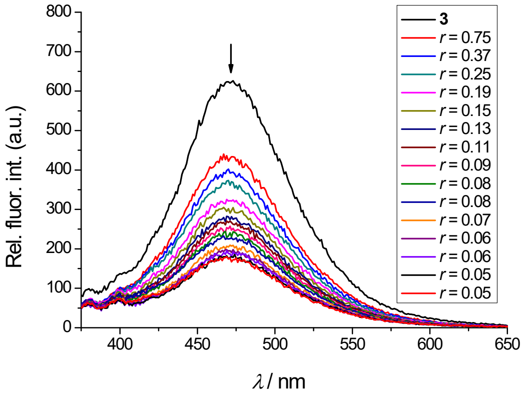

c)

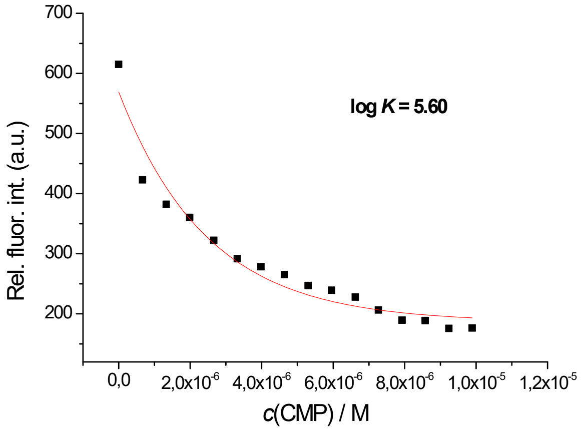

d)

Figure S23. a) Fluorimetric titration of **2** (c = 5 × 10^{-7} M; λ_{exc}= 350 nm) with CMP (c = 1 × 10^{-3} M) and b) dependence of fluorescence at λ_{max}= 475 nm on c(CMP); c) fluorimetric titration of **3** (c = 5 × 10^{-7} M; λ_{exc}= 350 nm) with CMP (c = 1 × 10^{-3} M) and d) dependence of fluorescence at λ_{max}= 475 nm on c(CMP). Done in sodium cacodylate buffer (pH=7.0, I = 0.05 M); r = [compound] / [mononucleotide].

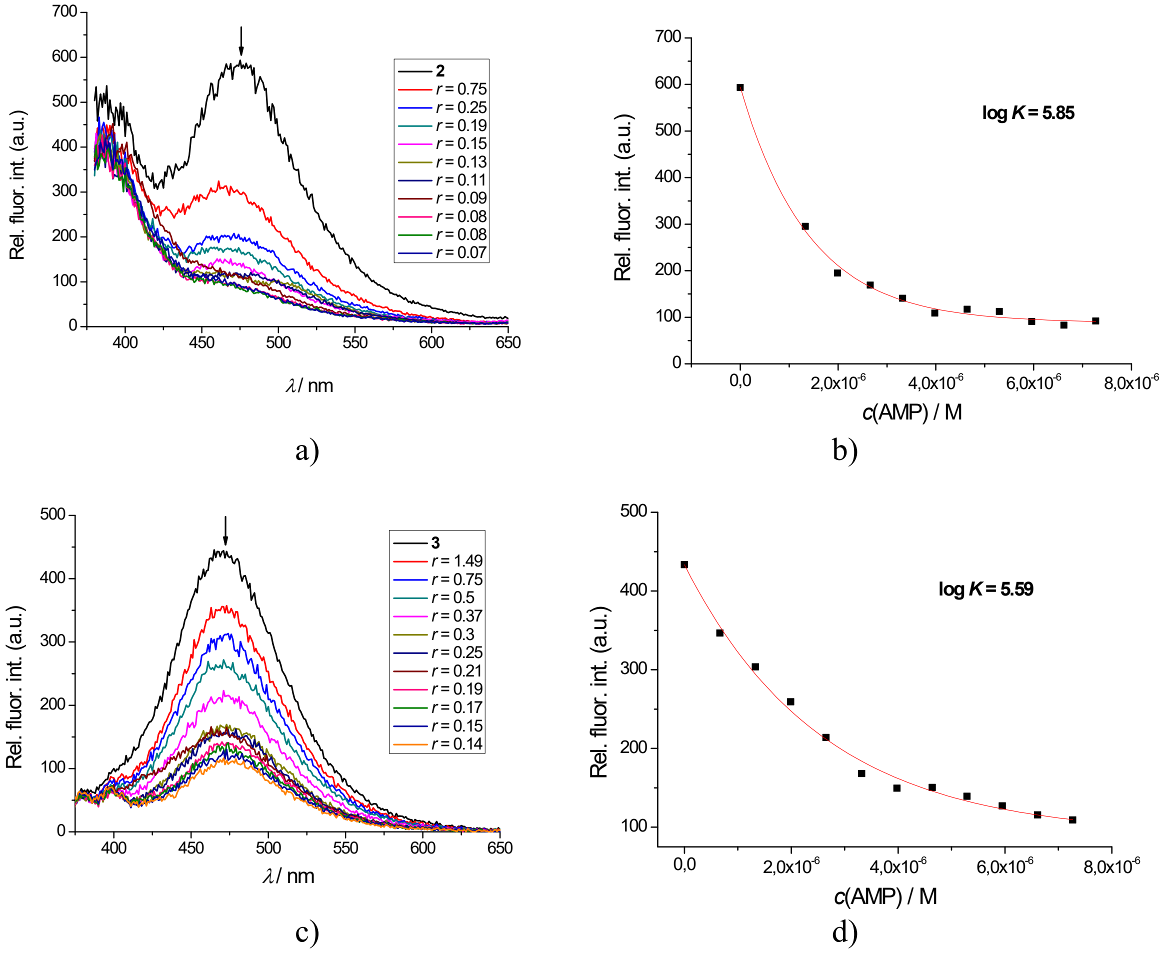

Figure S24. a) Fluorimetric titration of **2** (c = 5 × 10^{-7} M; λ_{exc}= 350 nm) with AMP (c = 1 × 10^{-3} M) and b) dependence of fluorescence at λ_{max}= 475 nm on c(AMP); c) fluorimetric titration of **3** (c = 1 × 10^{-6} M; λ_{exc}= 350 nm) with AMP (c = 1 × 10^{-3} M) and d) dependence of fluorescence at λ_{max}= 475 nm on c(AMP). Done in sodium cacodylate buffer (pH=7.0, I = 0.05 M); r = [compound] / [mononucleotide].

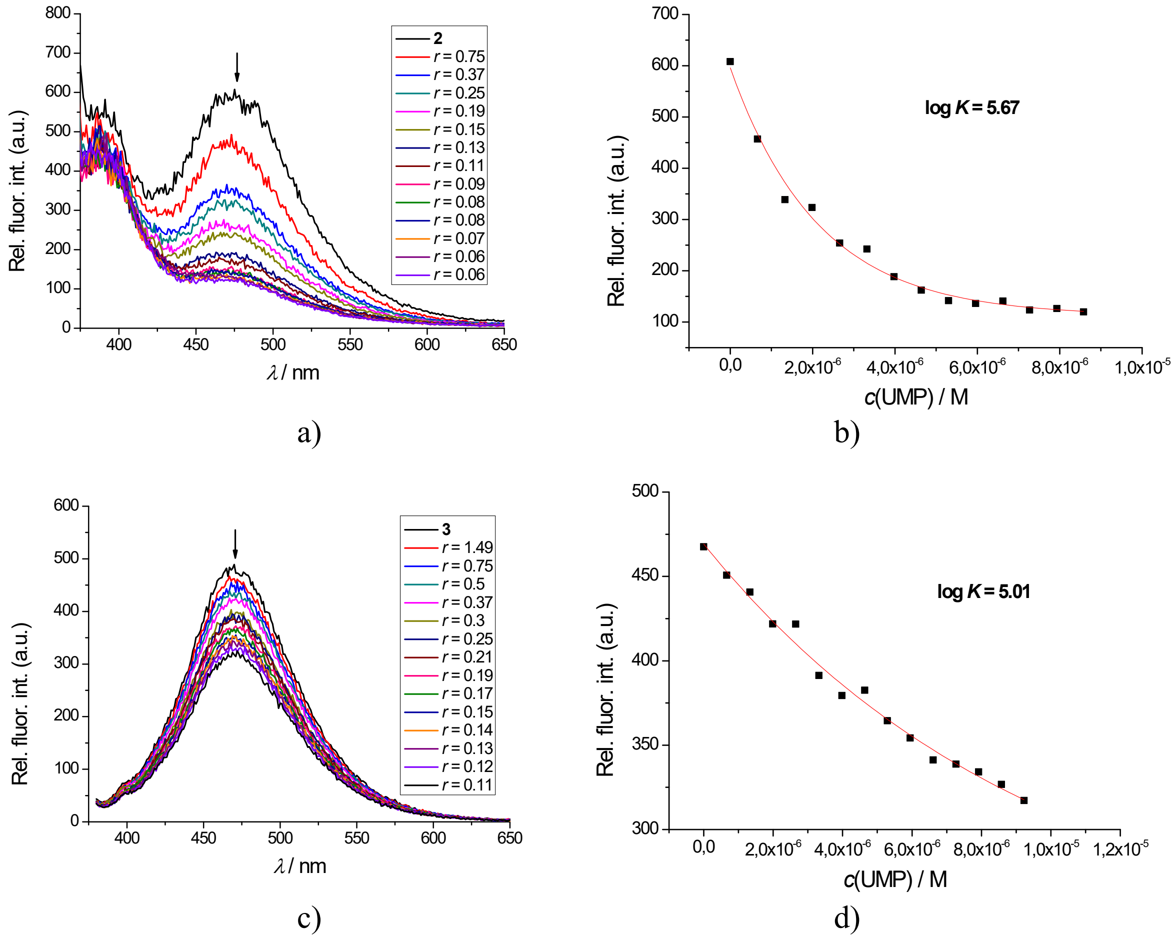

Figure S25. a) Fluorimetric titration of **2** (c = 5 × 10^{-7} M; λ_{exc}= 350 nm) with UMP (c = 1 × 10^{-3} M) and b) dependence of fluorescence at λ_{max}= 475 nm on c(UMP); c) fluorimetric titration of **3** (c = 1 × 10^{-6} M; λ_{exc}= 350 nm) with UMP (c = 1 × 10^{-3} M) and d) dependence of fluorescence at λ_{max}= 475 nm on c(UMP). Done in sodium cacodylate buffer (pH=7.0, I = 0.05 M); r = [compound] / [mononucleotide].

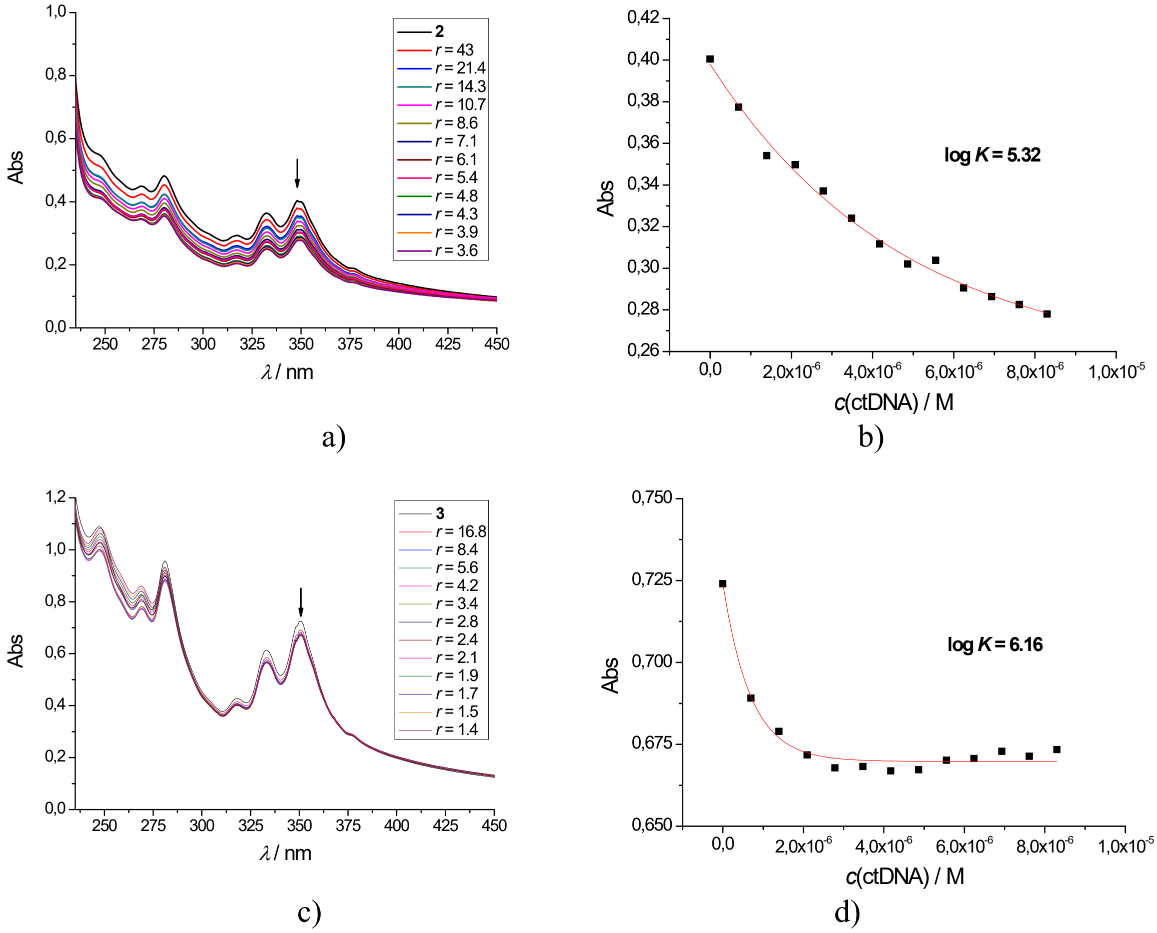

Figure S26. a) Spectrophotometric titration of **2** (c = 3 × 10^{-5} M) with ctDNA (c = 7 × 10^{-4} M) and b) dependence of absorption at λ_{max}= 350 nm on c(ctDNA); c) spectrophotometric titration of **3** (c = 3 × 10^{-5} M) with ctDNA (c = 1.79 × 10^{-3} M) and d) dependence of absorption at λ_{max}= 350 nm on c(ctDNA). Done in sodium cacodylate buffer (pH=7.0, I = 0.05 M); r = [compound] / [DNA/RNA].

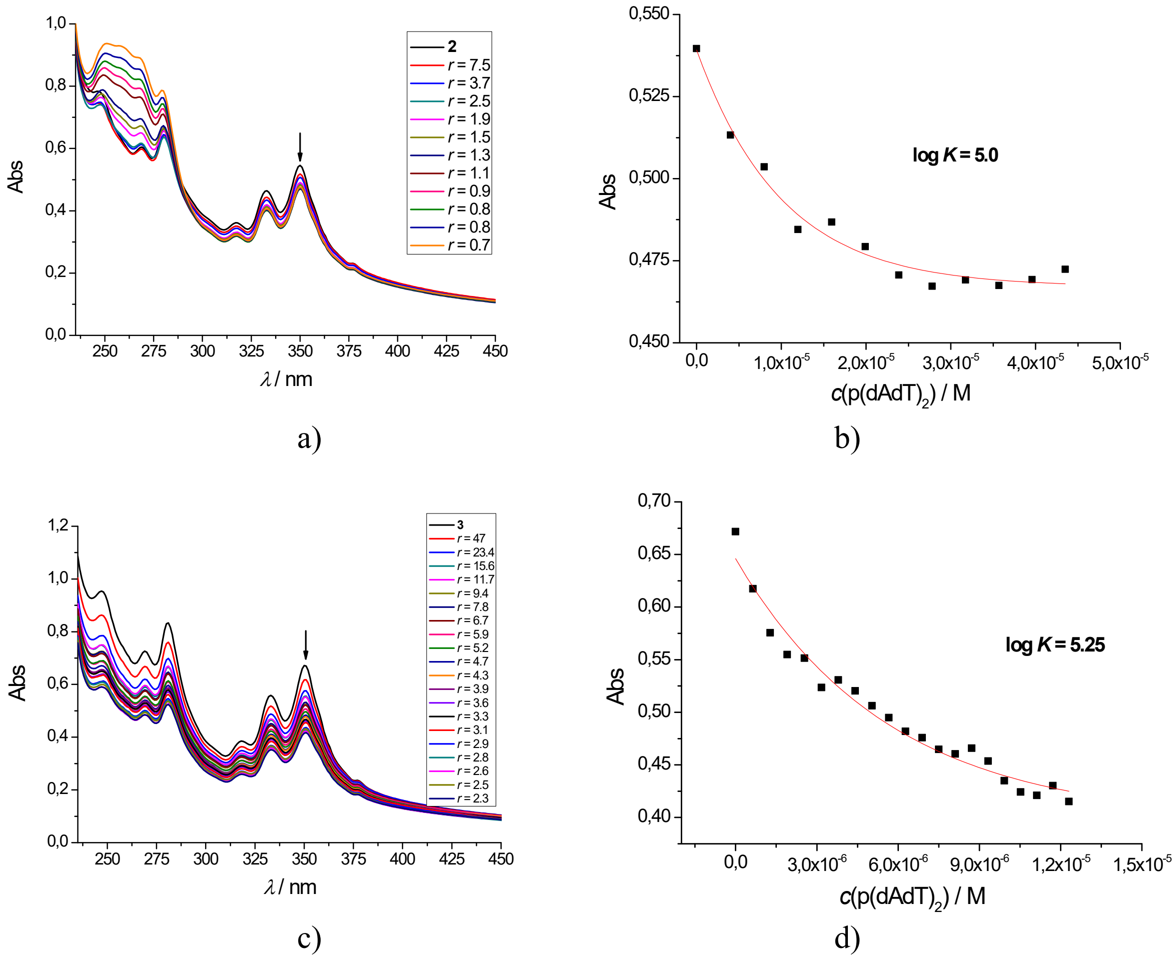

Figure S27. a) Spectrophotometric titration of **2** ($c = 3 \times 10^{-5}$ M) with p(dAdT)$_2$ ($c = 4 \times 10^{-3}$ M) and b) dependence of absorption at λ_{max}= 350 nm on c(p(dAdT)$_2$); c) spectrophotometric titration of **3** ($c = 3 \times 10^{-5}$ M) with p(dAdT)$_2$ ($c = 3.2 \times 10^{-4}$ M) and d) dependence of absorption at λ_{max}= 350 nm on c(p(dAdT)$_2$). Done in sodium cacodylate buffer (pH=7.0, I = 0.05 M); r = [compound] / [DNA/RNA].

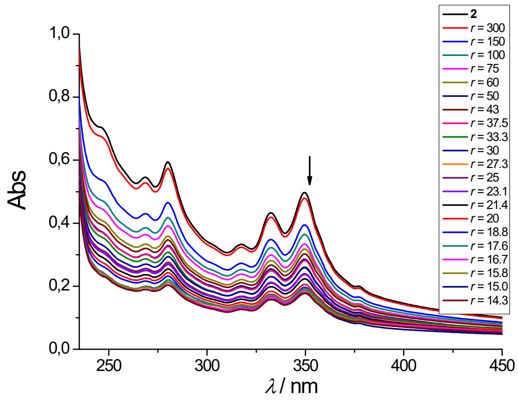

a)

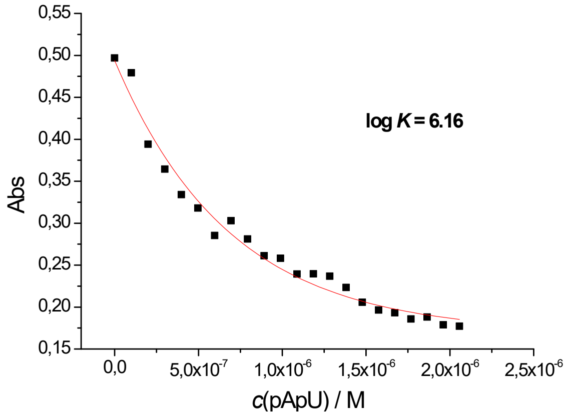

b)

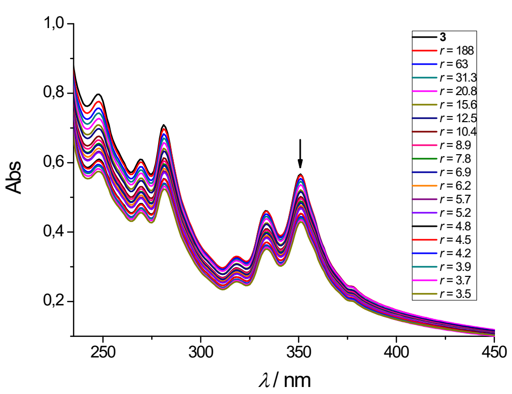

c)

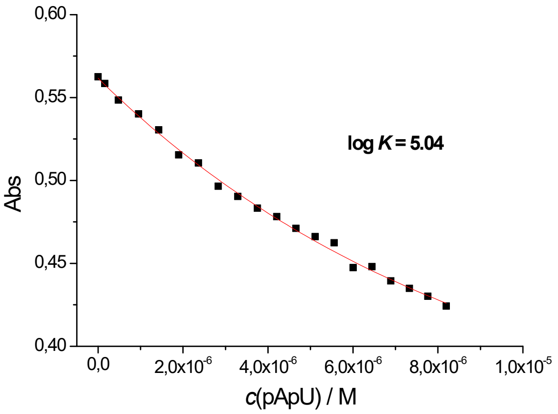

d)

Figure S28. a) Spectrophotometric titration of **2** ($c = 3 \times 10^{-5}$ M) with pApU ($c = 1 \times 10^{-4}$ M) and b) dependence of absorption at λ_{max}= 350 nm on c(pApU); c) spectrophotometric titration of **3** ($c = 3 \times 10^{-5}$ M) with pApU ($c = 1.6 \times 10^{-4}$ M) and d) dependence of absorption at λ_{max}= 350 nm on c(pApU). Done in sodium cacodylate buffer (pH=7.0, I = 0.05 M); r = [compound] / [DNA/RNA].

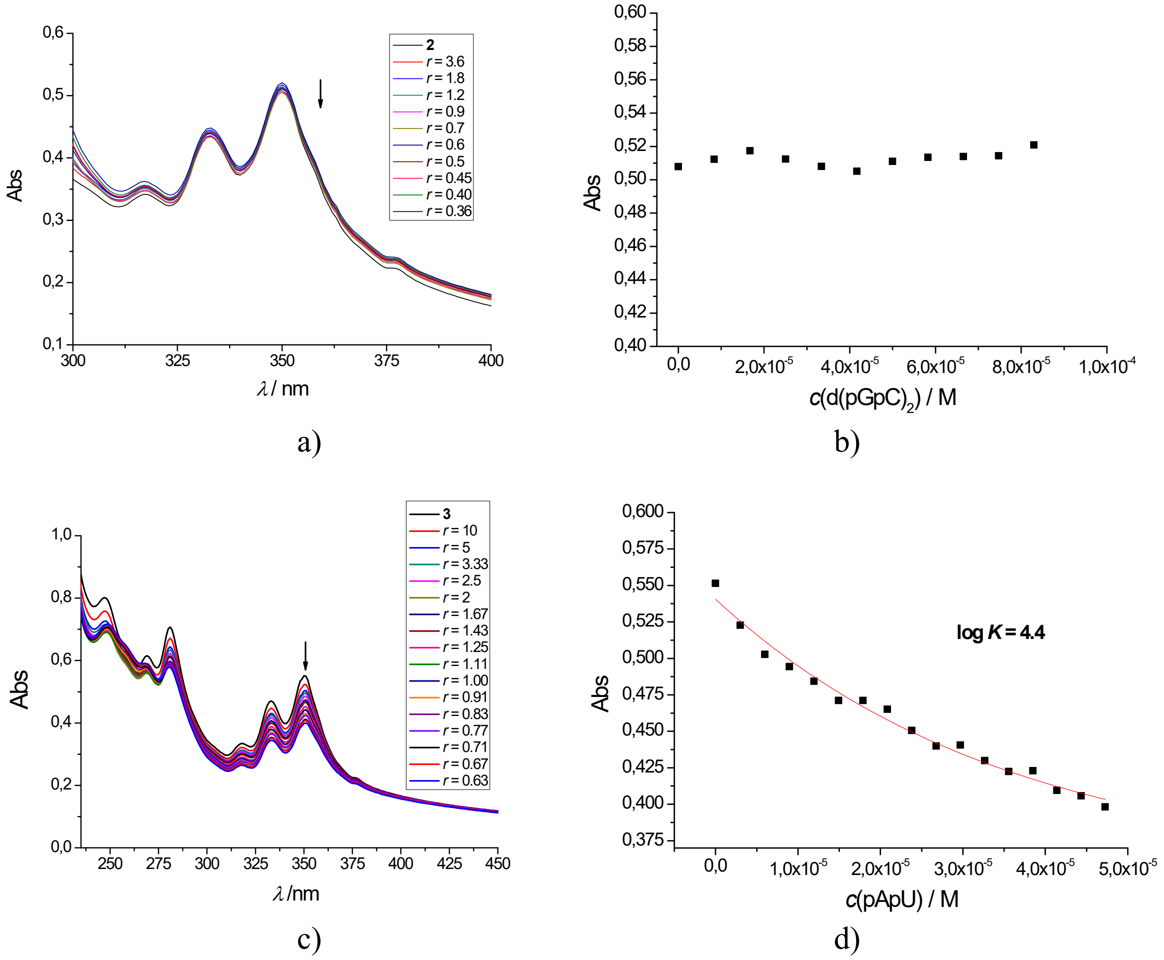

Figure S29. a) Spectrophotometric titration of **2** ($c = 3 \times 10^{-5}$ M) with p(dGdC)$_2$ ($c = 8.38 \times 10^{-3}$ M) and b) dependence of absorption at λ_{max}= 350 nm on c(p(dGdC)$_2$); c) spectrophotometric titration of **3** ($c = 3 \times 10^{-5}$ M) with p(dGdC)$_2$ ($c = 3 \times 10^{-3}$ M) and d) dependence of absorption at λ_{max}= 350 nm on c(p(dGdC)$_2$). Done in sodium cacodylate buffer (pH=7.0, I = 0.05 M); r = [compound] / [DNA/RNA].

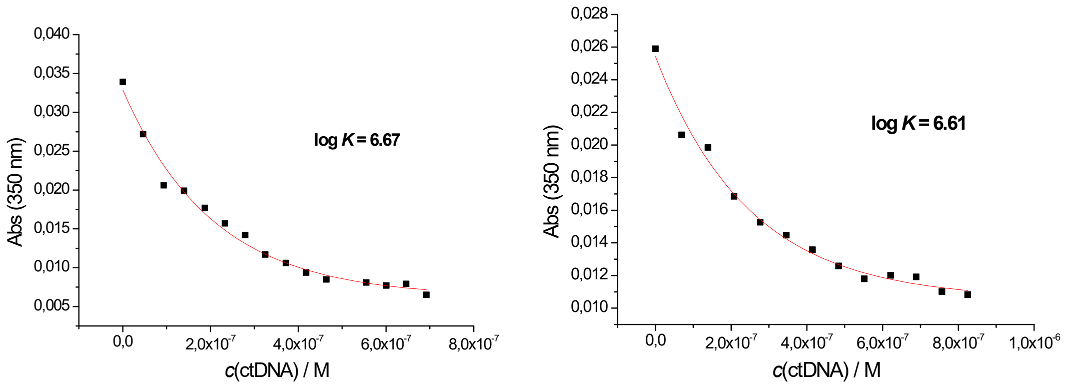

a) b)

Figure S30. a) Absorption dependence of **2** (c = 2 × 10^{-6} M) on concentration of ctDNA (c = 4.7 × 10^{-5} M) at λ_{max}= 350 nm; b) Absorption dependence of **3** (c = 3 × 10^{-6} M) on concentration of ctDNA (c = 7 × 10^{-5} M) at λ_{max}= 350 nm on c(ctDNA). Done in sodium cacodylate buffer (pH=7.0, I = 0.05 M); r = [compound] / [DNA/RNA].

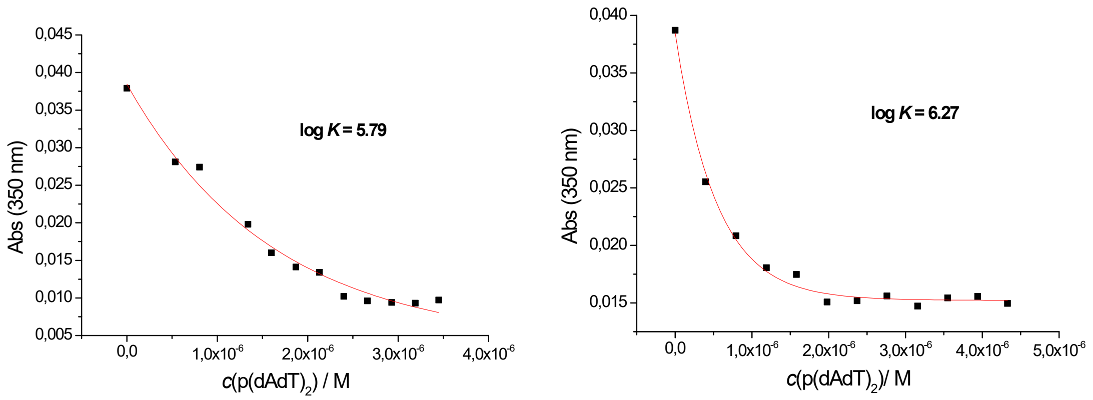

a) b)

Figure S31. a) Absorption dependence of **2** (c = 2 × 10^{-6} M) on concentration of p(dAdT)$_2$ (c = 2.7 × 10^{-4} M) at λ_{max}= 350 nm on c(p(dAdT)$_2$); b) Absorption dependence of **3** (c = 3 × 10^{-6} M) on concentration of p(dAdT)$_2$ (c = 4 × 10^{-4} M) at λ_{max}= 350 nm on c(p(dAdT)$_2$). Done in sodium cacodylate buffer (pH=7.0, I = 0.05 M); r = [compound] / [DNA/RNA].

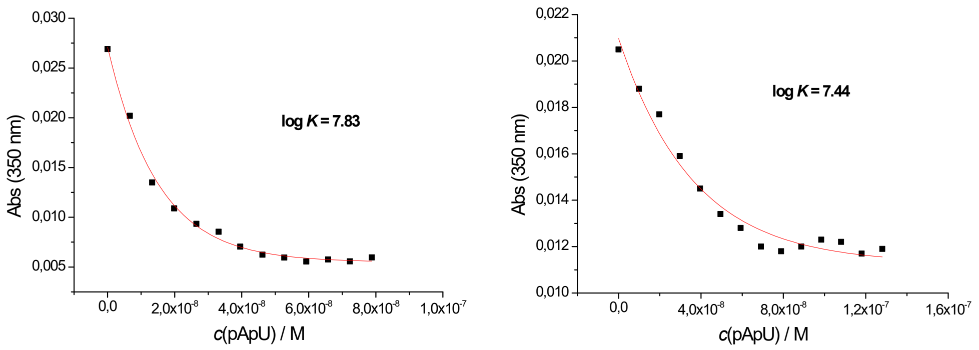

a) b)

Figure S32. a) Absorption dependence of **2** (c = 2 × 10^{-6} M) on concentration of pApU (c = 6.67 × 10^{-6} M) at λ_{max}= 350 nm on c(pApU); b) Absorption dependence of **3** (c = 3 × 10^{-6} M) on concentration pApU (c = 1 × 10^{-5} M) at λ_{max}= 350 nm on c(pApU). Done in sodium cacodylate buffer (pH=7.0, I = 0.05 M); r = [compound] / [DNA/RNA].

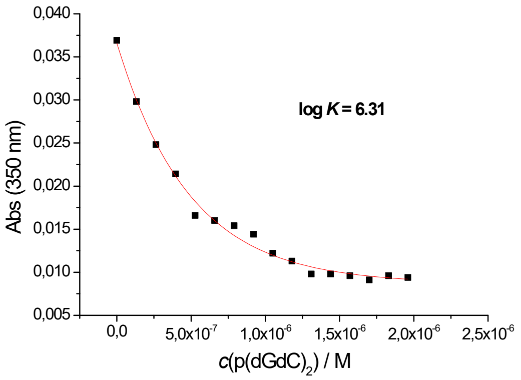

a)

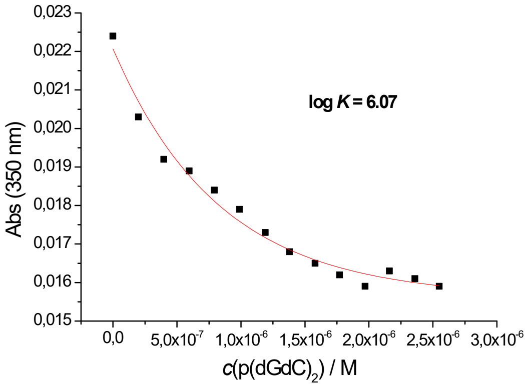

b)

Figure S33. a) Absorption dependence of **2** ($c = 2 \times 10^{-6}$ M) on concentration of p(dGdC)$_2$ ($c = 1.33 \times 10^{-4}$ M) at λ_{max}= 350 nm on c(p(dGdC)$_2$); b) Absorption dependence of **3** ($c = 3 \times 10^{-6}$ M) on concentration of p(dGdC)$_2$ ($c = 2 \times 10^{-4}$ M) at λ_{max}= 350 nm on c(p(dGdC)$_2$). Done in sodium cacodylate buffer (pH=7.0, I = 0.05 M); r = [compound] / [DNA/RNA].

Table S2. Binding constants ([a]log$K$) for **2** and **3** with mononucleotides and ds-DNA/ds-RNA determined spectrophotometrically. Done in sodium cacodylate buffer (pH = 7.0, I = 0.05 M).

	**2**	**3**
CMP	5.4	5.4
AMP	5.0	5.2
UMP	5.1	5.0
GMP	5.2	5.6
ctDNA	6.7[b]	6.6[b]
p(dAdT)$_2$	5.8[b]	6.3[b]
pApU	7.8[b]	7.4[b]
p(dGdC)$_2$	6.3[b]	6.1[b]

[a] The best fit of experimental data was obtained for 1:1 stoichiometry of dye/NMP complex or for DNA/RNA by processing of titration data by means of Scatchard equation gave values of ratio n[bound dye]/ [DNA/RNA] = 0.1 and 0.2, for easier comparison all log$K$ values were re-calculated for fixed n = 0.2.

[b] UV-Vis titration performed at 350 nm.

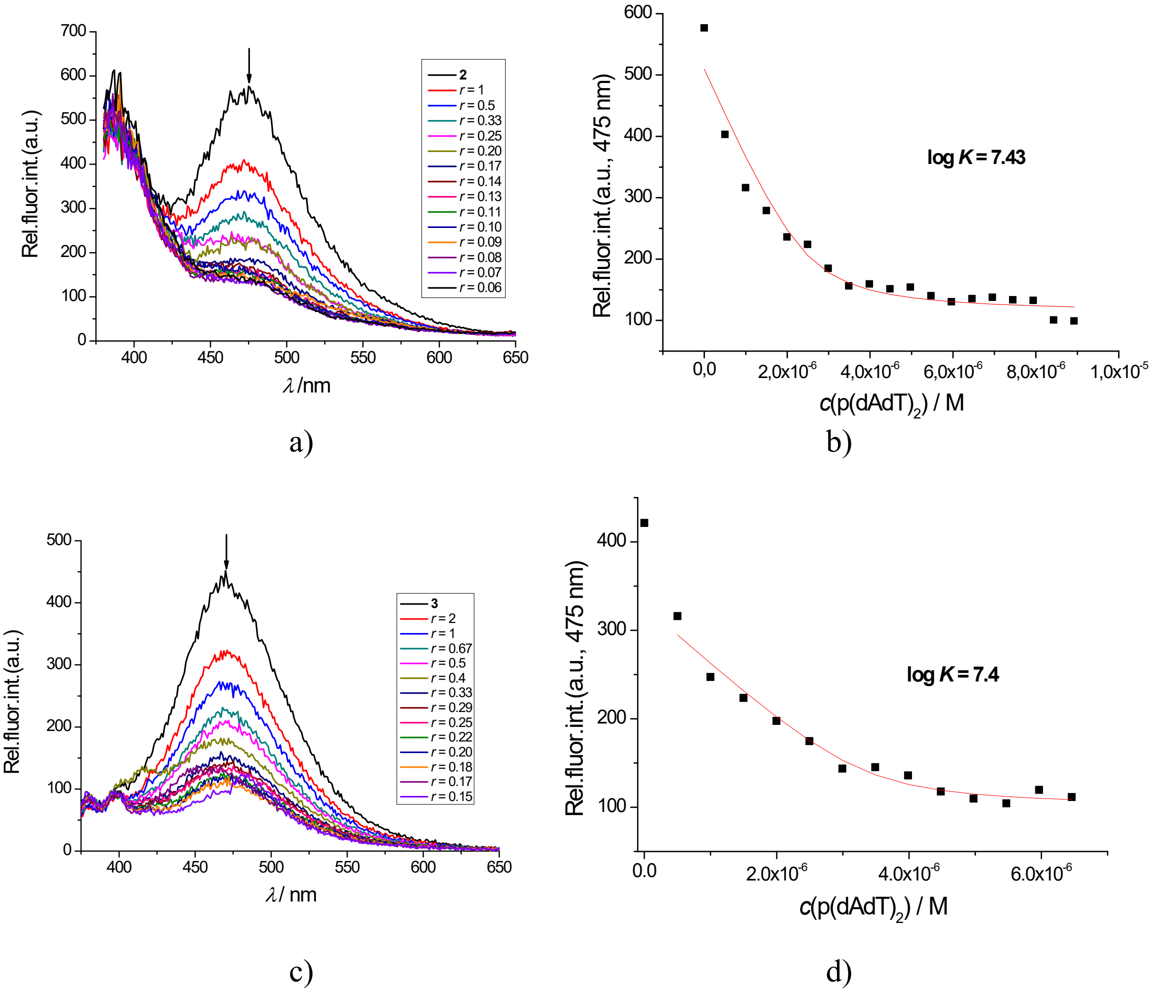

Figure S34. a) Fluorimetric titration of **2** ($c = 5 \times 10^{-7}$ M; λ_{exc}= 350 nm) with p(dAdT)$_2$ ($c = 1 \times 10^{-3}$ M) and b) dependence of fluorescence at λ_{max}= 475 nm on c(p(dAdT)$_2$); c) fluorimetric titration of **3** ($c = 1 \times 10^{-6}$ M; λ_{exc}= 350 nm) with p(dAdT)$_2$ ($c = 1 \times 10^{-3}$ M) and d) dependence of fluorescence at λ_{max}= 475 nm on c(p(dAdT)$_2$). Done in sodium cacodylate buffer (pH=7.0, I = 0.05 M); r = [compound] /[DNA/RNA].

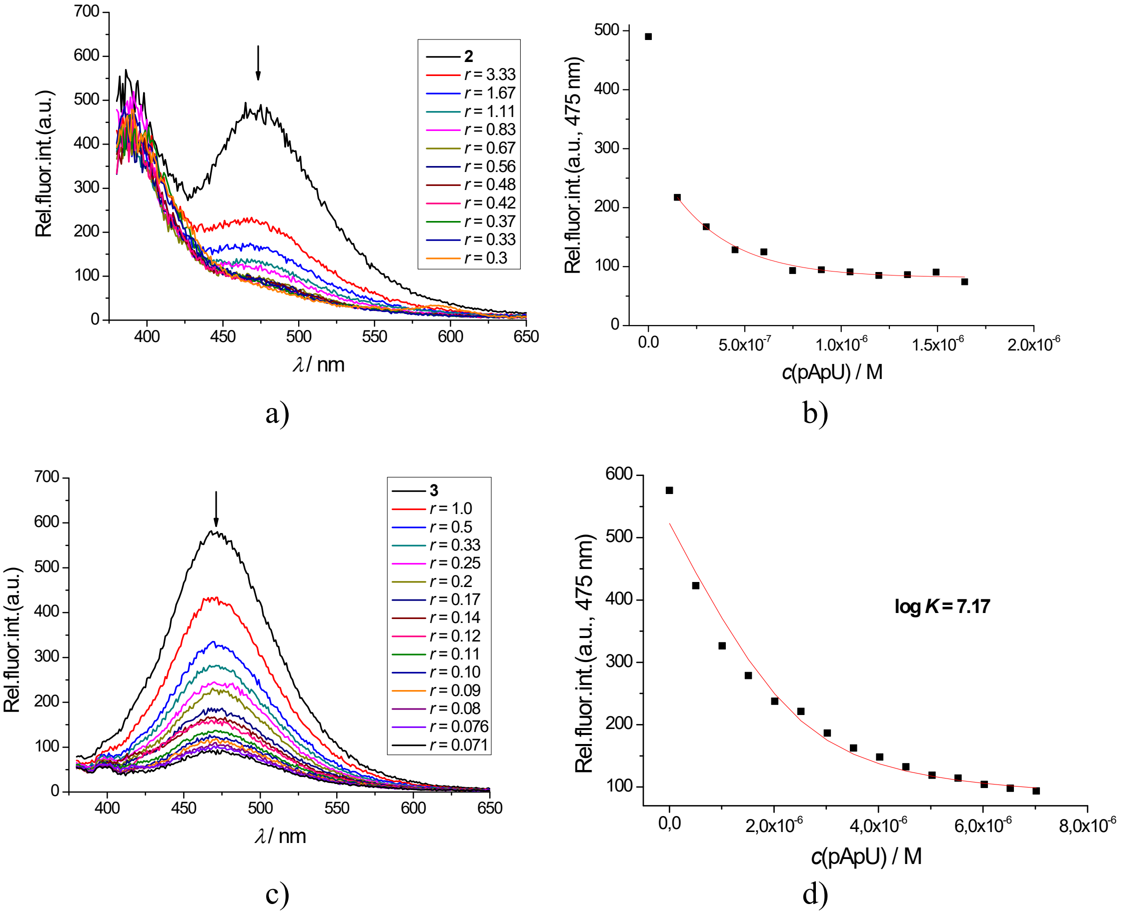

Figure S35. a) Fluorimetric titration of **2** ($c = 5 \times 10^{-7}$ M; λ_{exc}= 350 nm) with pApU ($c = 3 \times 10^{-4}$ M) and b) dependence of fluorescence at λ_{max}= 475 nm on *c*(pApU); c) fluorimetric titration of **3** ($c = 5 \times 10^{-7}$ M; λ_{exc}= 350 nm) with pApU ($c = 1.01 \times 10^{-3}$ M) and d) dependence of fluorescence at λ_{max}= 475 nm on *c*(pApU). Done in sodium cacodylate buffer (pH=7.0, I = 0.05 M); r = [compound] / [DNA/RNA].

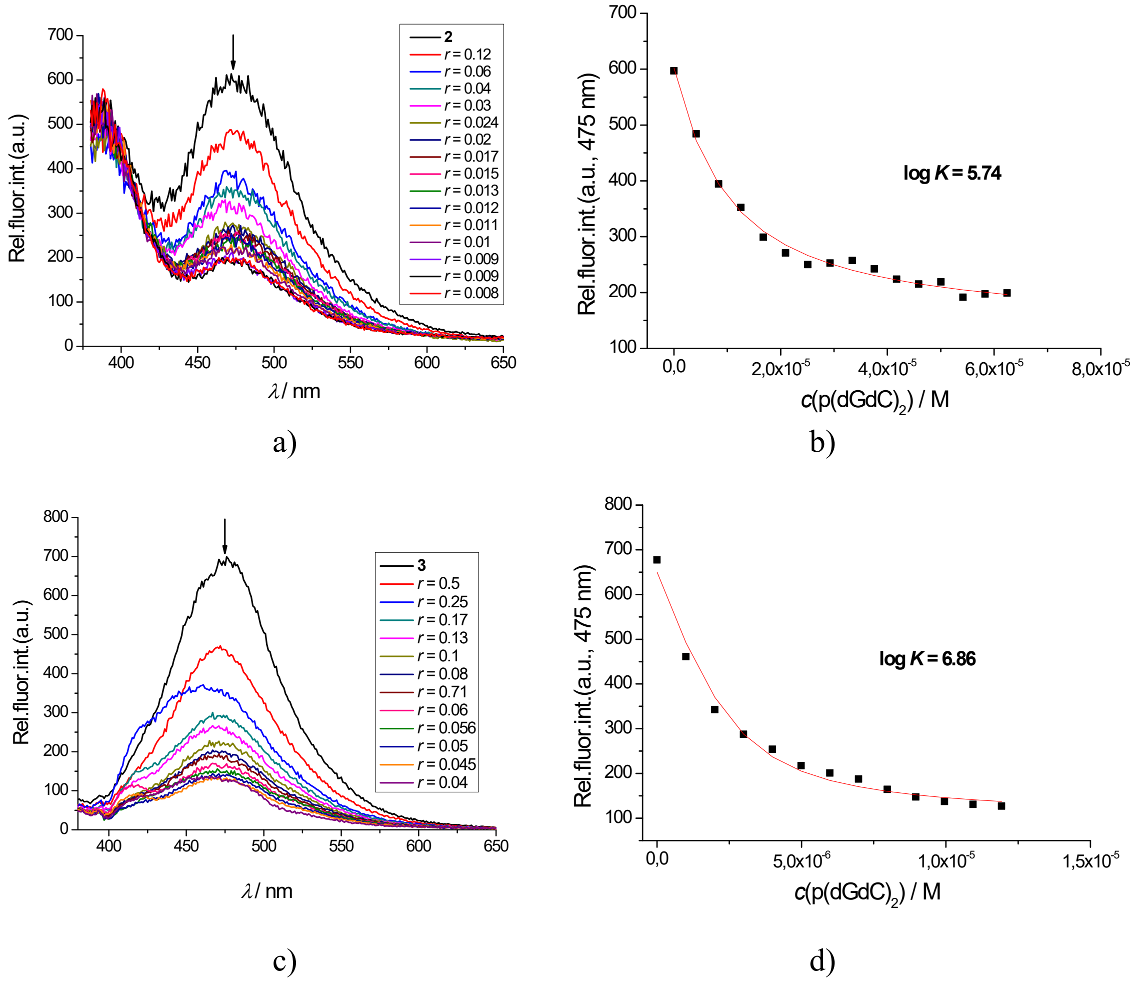

Figure S36. a) Fluorimetric titration of **2** (c = 5 × 10^{-7} M; λ_{exc}= 350 nm) with p(dGdC)$_2$ (c = 8.39 × 10^{-3} M) and b) dependence of fluorescence at λ_{max}= 475 nm on c(p(dGdC)$_2$); c) fluorimetric titration of **3** (c = 5 × 10^{-7} M; λ_{exc}= 350 nm) with p(dGdC)$_2$ (c = 2 × 10^{-3} M) and d) dependence of fluorescence at λ_{max}= 475 nm on c(p(dGdC)$_2$). Done in sodium cacodylate buffer (pH=7.0, I = 0.05 M); r = [compound] / [DNA/RNA].

3.2. Circular dichroism (CD) experiments

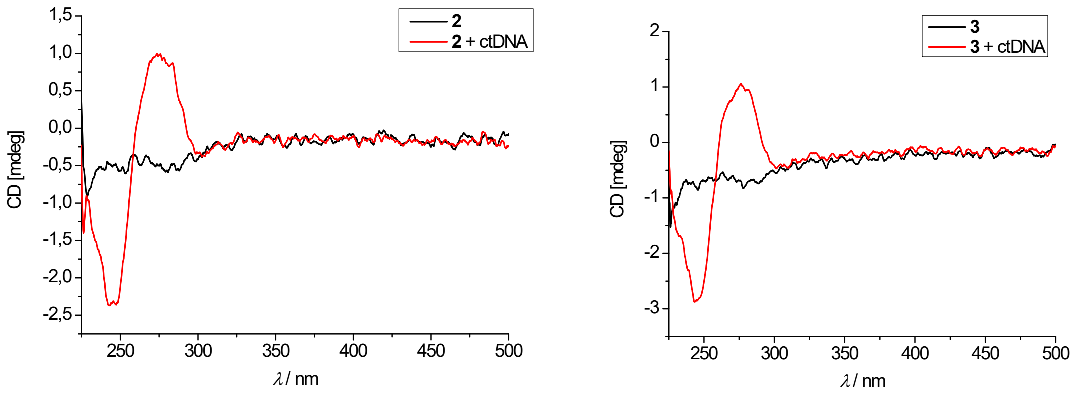

a) b)

Figure S37. CD spectra of: a) **2** and b) **3** ($c = 1 \times 10^{-5}$ M) and after addition of ctDNA ($c = 3$ x 10^{-5} M) at molar ratio $r = 3$ (r = [compound] / [polynucleotide]). Done at pH = 7.0, sodium cacodylate buffer, $I = 0.05$ M.

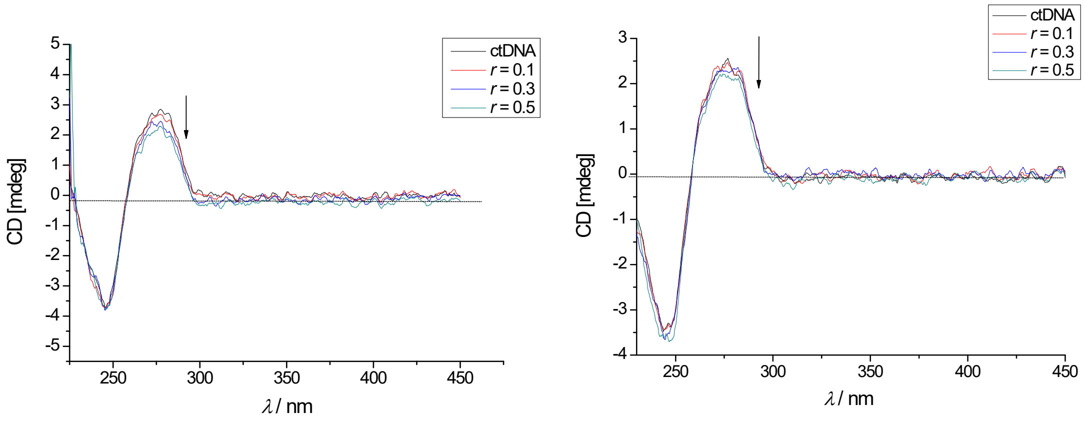

a) b)

Figure S38. CD titration of ctDNA ($c = 3 \times 10^{-5}$ M) with: a) **2** ($c = 5 \times 10^{-3}$ M) and b) **3** ($c = 5 \times 10^{-3}$ M) at different ratio. Done in sodium cacodylate buffer (pH = 7.0, $I = 0.05$ M); r = [compound] / [DNA/RNA].

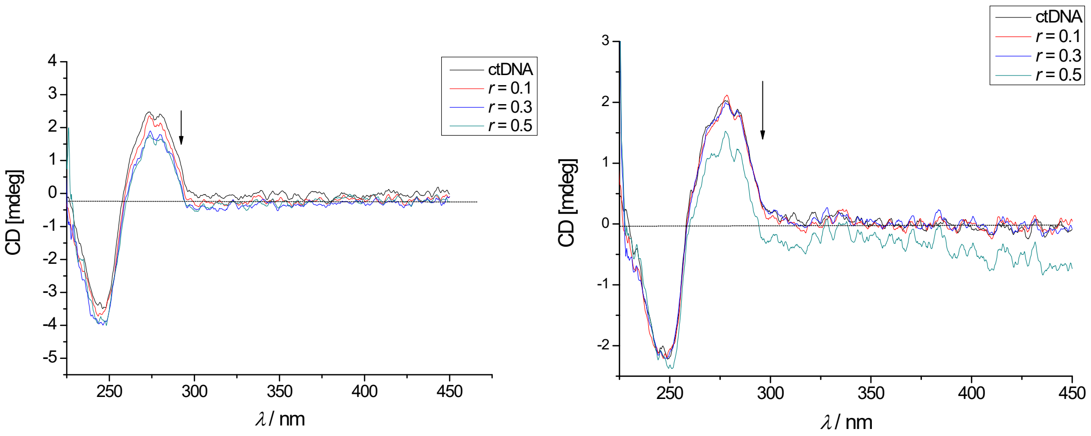

a) b)

Figure S39. CD titration of ctDNA (c = 3 × 10^{-5} M) with: a) **2**/Cu^{2+} (c = 1 × 10^{-3} M) and b) **3**/Cu^{2+} (c = 1 × 10^{-3} M) at different ratio. Done in sodium cacodylate buffer (pH = 7.0, I = 0.05 M); r = [compound] / [DNA/RNA].

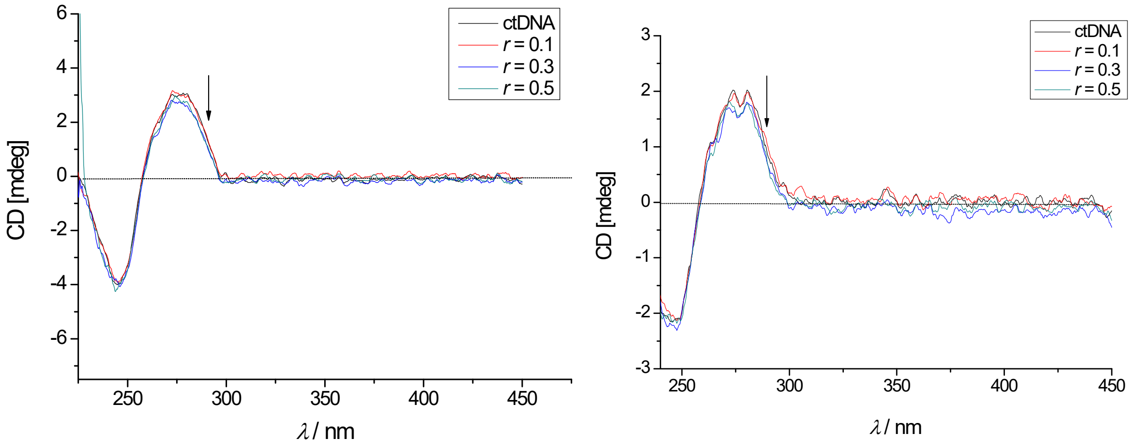

a) b)

Figure S40. CD titration of ctDNA (c = 3 × 10^{-5} M) with: a) **2**/Zn^{2+} (c = 1 × 10^{-3} M) and b) **3**/Zn^{2+} (c = 1 × 10^{-3} M) at different ratio. Done in sodium cacodylate buffer (pH = 7.0, I = 0.05 M); r = [compound] / [DNA/RNA].

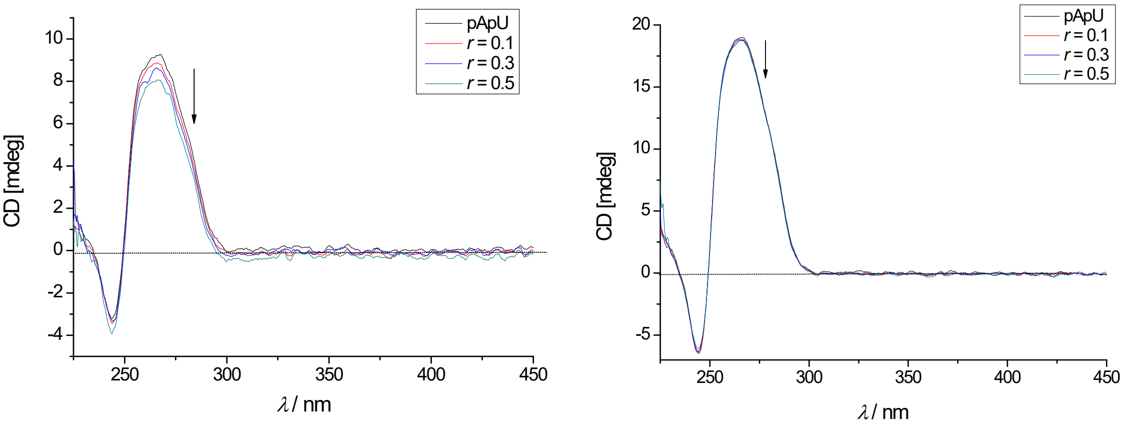

a) b)

Figure S41. CD titration of pApU ($c = 3 \times 10^{-5}$ M) with: a) **2** ($c = 5 \times 10^{-3}$ M) and b) **3** ($c = 5 \times 10^{-3}$ M) at different ratio. Done in sodium cacodylate buffer (pH = 7.0, $I = 0.05$ M); r = [compound] / [DNA/RNA].

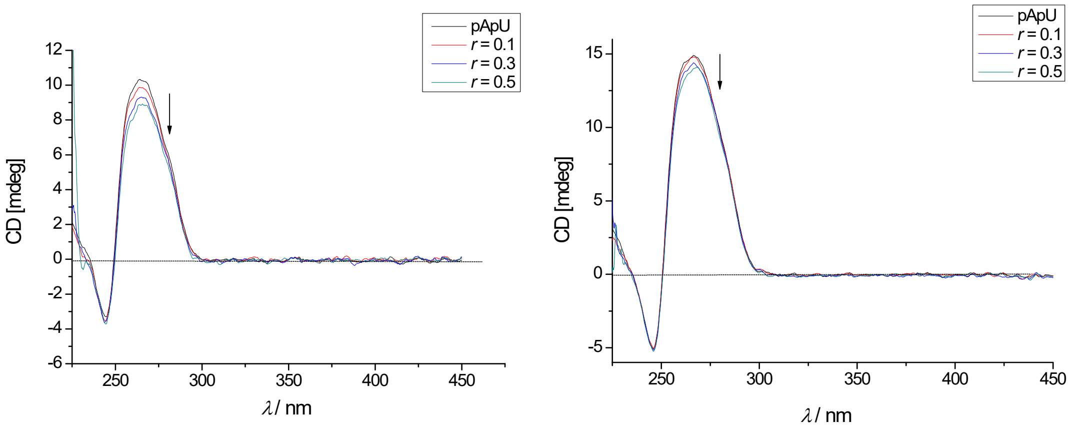

a) b)

Figure S42. CD titration of pApU ($c = 3 \times 10^{-5}$ M) with: a) **2**/Cu^{2+} ($c = 5 \times 10^{-3}$ M) and b) **3**/Cu^{2+} ($c = 5 \times 10^{-3}$ M) at different ratio. Done in sodium cacodylate buffer (pH = 7.0, $I = 0.05$ M); r = [compound] / [DNA/RNA].

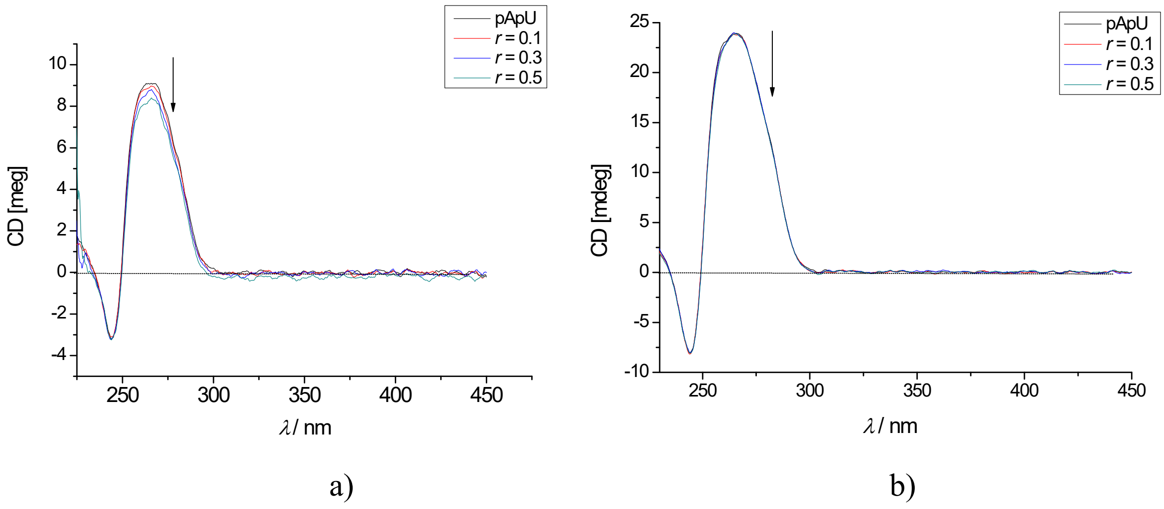

Figure S43. CD titration of pApU ($c = 3 \times 10^{-5}$ M) with: a) **2**/Zn^{2+} ($c = 5 \times 10^{-3}$ M) and b) **3**/Zn^{2+} ($c = 5 \times 10^{-3}$ M) at different ratio. Done in sodium cacodylate buffer (pH = 7.0, $I = 0.05$ M); r = [compound] / [DNA/RNA].

3.3. Thermal denaturation experiments

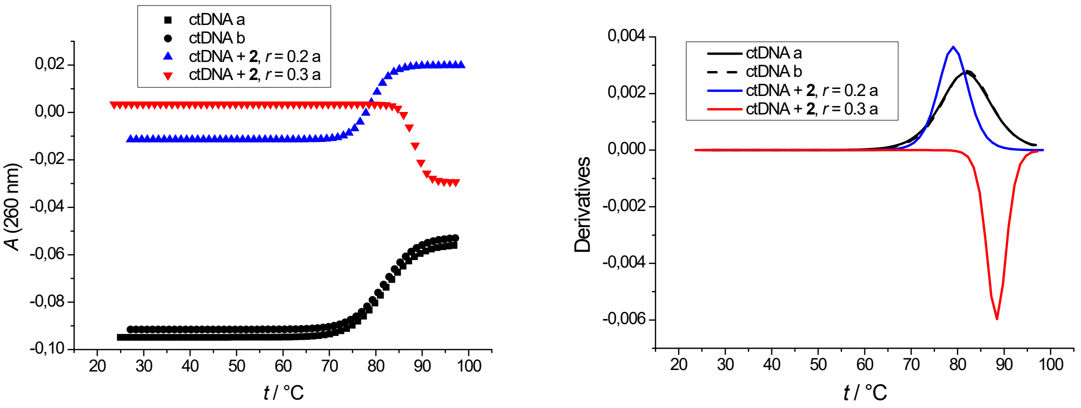

a)

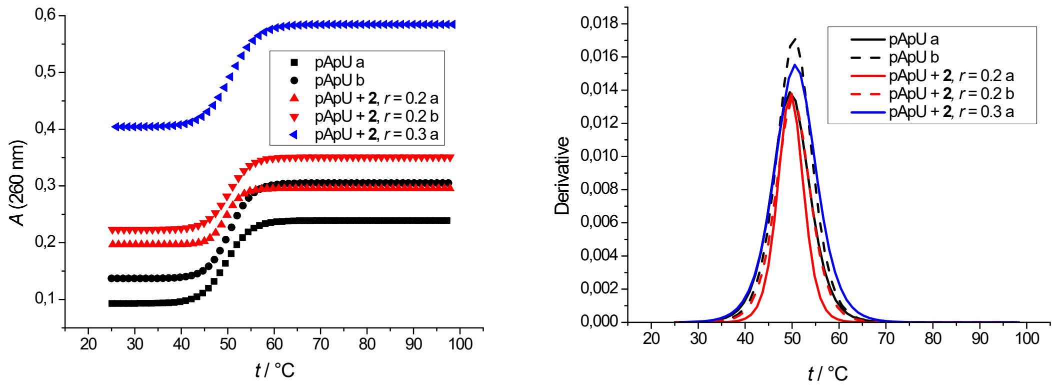

b)

Figure S44. Thermal denaturation curves (left) and derivatives (right) of: a) ctDNA (c(ctDNA) = 2.27 × 10^{-5} M) and b) pApU (c(pApU) = 2.27 × 10^{-5} M) with **2** (c(**2**) = 5 × 10^{-3} M). Done in sodium cacodylate buffer (pH=7.0, I = 0.05 M); r = [compound] / [DNA/RNA].

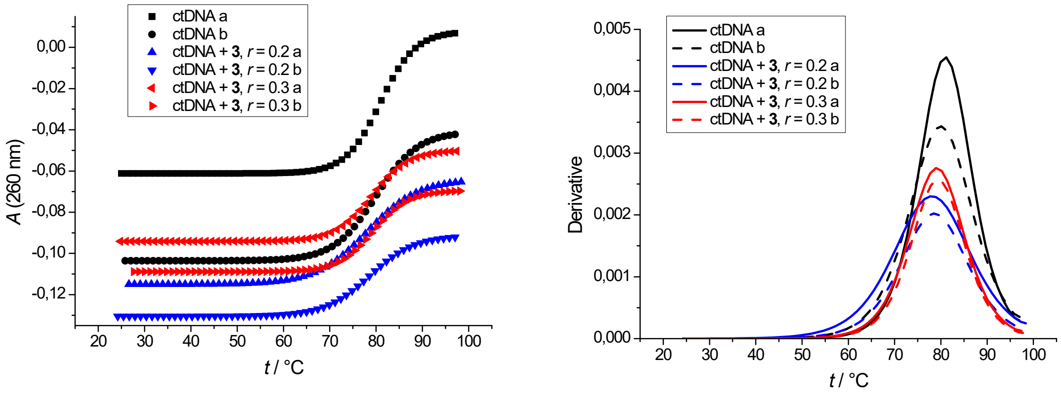

a)

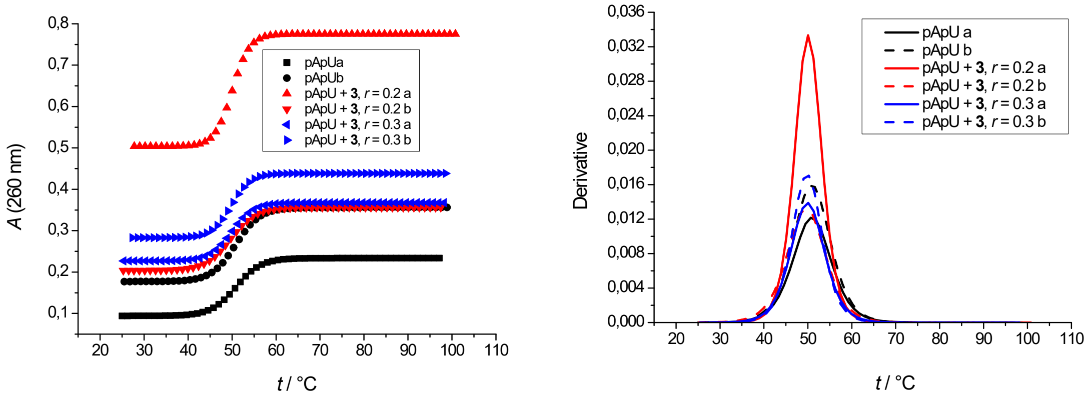

b)

Figure S45. Thermal denaturation curves (left) and derivatives (right) of: a) ctDNA (c(ctDNA) = 2.27×10^{-5} M) and b) pApU (c(pApU) = 2.27×10^{-5} M) with **3** (c(**3**) = 5×10^{-3} M). Done in sodium cacodylate buffer (pH=7.0, I = 0.05 M); r = [compound] / [DNA/RNA].

Table S3. ΔT_m values for ctDNA and pApU with **2** and **3** at different ratios. Done in sodium cacodylate buffer (pH=7.0, I = 0.05 M); r = [compound] / [DNA/RNA].

		**ctDNA**	**pApU**
**2**	r = 0.2	-2	0
	r = 0.3	-	+1
**3**	r = 0.2	-2	-1
	r = 0.3	-1	-1

$\Delta T_m = T_{m\ poly+compound} - T_{m\ poly}$
